# Histone Deacetylase Inhibitors as Multitarget-Directed Epi-Drugs in Blocking PI3K Oncogenic Signaling: A Polypharmacology Approach

**DOI:** 10.3390/ijms21218198

**Published:** 2020-11-02

**Authors:** Kasturi Ranganna, Chelliah Selvam, Amruthesh Shivachar, Zivar Yousefipour

**Affiliations:** Department of Pharmaceutical Science, College of Pharmacy and Health Sciences, Texas Southern University, 3100 Cleburne St., Houston, TX 77004, USA; Selvam.chelliah@tsu.edu (C.S.); Amruthesh.Shivachar@tsu.edu (A.S.); Zivar.Yousefipour@tsu.edu (Z.Y.)

**Keywords:** epigenetics, histone deacetylase, histone deacetylase inhibitors, phosphatidylinositol kinase, HDAC-PI3K hybrid molecule, CUDC-907

## Abstract

Genetic mutations and aberrant epigenetic alterations are the triggers for carcinogenesis. The emergence of the drugs targeting epigenetic aberrations has provided a better outlook for cancer treatment. Histone deacetylases (HDACs) are epigenetic modifiers playing critical roles in numerous key biological functions. Inappropriate expression of HDACs and dysregulation of PI3K signaling pathway are common aberrations observed in human diseases, particularly in cancers. Histone deacetylase inhibitors (HDACIs) are a class of epigenetic small-molecular therapeutics exhibiting promising applications in the treatment of hematological and solid malignancies, and in non-neoplastic diseases. Although HDACIs as single agents exhibit synergy by inhibiting HDAC and the PI3K pathway, resistance to HDACIs is frequently encountered due to activation of compensatory survival pathway. Targeted simultaneous inhibition of both HDACs and PI3Ks with their respective inhibitors in combination displayed synergistic therapeutic efficacy and encouraged the development of a single HDAC-PI3K hybrid molecule via polypharmacology strategy. This review provides an overview of HDACs and the evolution of HDACs-based epigenetic therapeutic approaches targeting the PI3K pathway.

## 1. Introduction

Phosphatidylinositol 3-kinases (PI3Ks) are a family of intracellular signaling lipid kinases that regulate a wide array of cellular processes essentially in all normal tissues. Members of the PI3Ks family are categorized into three distinct classes (class I-III) based on their structure, expression, regulation, and substrate specificity. Class I PI3K family includes well-known four different PI3Ks named PI3Kα, PI3Kβ, PI3Kγ, and PI3Kδ and are implicated in remarkably diverse pathways [1,2]. An extensive amount of research knowledge related to the PI3Ks reveals that they transduce a host of cellular signals and regulate a wide range of essential cellular processes and physiological functions, including cell growth, proliferation, differentiation, survival, migration, death, and metabolism [1,2,3]. Naturally, the PI3K pathway is highly regulated by multiple mechanisms, often involving crosstalk with other signaling pathways in various physiological and pathophysiological situations. Importantly, the major outcome of intense investigations of PI3K signaling is the realization that aberrant activation of signaling is strongly linked to human diseases particularly in various types of cancers [4,5,6,7]. In recent years, a number of PI3K pathway inhibitors have been developed using the PI3K pathway as a drug target in human cancers, and many of them are being used in preclinical and clinical trials. However, clinical trials with early versions of PI3K inhibitors (PI3KIs) used as single-target drugs in monotherapy have exhibited limited efficacy, due to compensatory or concurrent activation of other survival and growth-related signaling pathways [8,9].

To overcome the limitations of a single-target approach, alternative strategies are being developed to block tumor growth and progression. Among these, inhibition of histone deacetylase (HDAC) activity appears to be a promising approach. By inducing growth-inhibitory cellular effects and by regulating both histones and non-histones substrates via regulating signaling pathways, histone deacetylase inhibitors (HDACIs) exhibit the potential to induce synergistic antitumor activity [10,11,12,13]. Moreover, the latest polypharmacology approach that is designed to overcome the limitations of a single-target approach appears to have a superior therapeutic effect with an affiliated reduction in adverse reactions and diminished potential drug resistance. Specifically, the epigenetic polypharmacology approach, using a dual-acting HDAC and PI3K inhibitor, by incorporating HDACI functionality into a PI3KI pharmacophore, is capable of potent anticancer activity [14,15,16]. Simultaneous inhibition of HDACs and PI3K activity by the dual HDAC and PI3K inhibitor can disrupt the oncogenic signaling network in cancer cells [14,15,16]. This review will focus on the role of HDACs and PI3K signaling in malignancy. Extend this information to understand the effectiveness of polypharmacology-based strategy in blocking oncogenic signaling networks.

## 2. Phosphatidylinositol 3-Kinases (PI3Ks)

### 2.1. Class I Phospatidylinositol 3-Kinases

Class I PI3Ks are involved in diverse pathways activated by an array of signals including growth factors, mitogenic factors, and hormones [1,2]. Class I PI3Ks are different in their tissue distribution and functions. While PI3Kα and PI3Kβ isoforms are ubiquitously expressed, PI3Kγ and PI3Kδ isoforms are enriched in hematopoietic cells such as leukocytes [17]. Class I PI3Ks are further subdivided into class IA (PI3Kα, PI3Kβ, and PI3Kδ) and class IB (PI3Kγ) and are heterodimers composed of a regulatory subunit (p85 or p101 family) and a catalytic subunit (p110α, p110β, p110γ or p110δ). All four class I catalytic subunits, p110α, p110β, p110γ, and p110δ are encoded by PIK3CA, PIK3CB, PIK3CG, and PIK3CD genes, respectively. The p85 regulatory subunit consists of p85α (p85α, p55α, or p50α splice variants), p85β and p85γ and is encoded by PIK3R1, PIk34R2, and PIKR3 genes, respectively. The catalytic subunits p110α, p110β, and p110δ associate with regulatory subunit p85 to form class IA PI3Kα, PI3Kβ, and PI3Kδ. The catalytic subunit p110γ and regulatory subunit p101 together form class IB PI3Kγ. All four class I PI3Ks catalyze the conversion of phosphatidylinositol 4,5-biphosphate [PI(4,5)P_2_] to phosphatidylinositol 3,4,5-triphosphate [PI(3,4,5,)P_3_]. PI(3,4,5,)P_3_ (PIP_3_) is a second messenger involved in the activation of a number of downstream molecules of the PI3K signaling pathway [1,2,18].

PI3Ks transduce a host of cellular signals and regulate a wide range of essential cellular processes and physiological functions, including cell growth, proliferation, differentiation, survival, migration, death, and metabolism [1,2,3]. Obviously, the PI3K pathway is highly regulated by multiple mechanisms, often involving crosstalk with other signaling pathways in various physiological and pathophysiological situations. Considering that the PI3K signaling is fundamental to normal cellular processes and physiological functions, glitches in the regulation of the PI3K pathway can lead to the pathogenesis of diseases. Overwhelming information indicates aberrant activation of the PI3K signaling is strongly linked to human cancers [4,5,6,7]. Non-cancerous neurological disorders [19,20], cardiovascular diseases [21,22,23], diabetes [24], immune disorders [17], and CLOVES syndrome characterized by localized tissue outgrowths [25] also share a common etiology with oncogenesis.

Abnormal mutational activation or amplification of the PIK3CA gene encoding p110α catalytic subunit of PI3Kα is the commonly observed event that is associated with a dysfunctional PI3K signaling cascade in different human solid and hematological malignancies [4,5,6,7]. The PI3K pathway is also dysregulated through other mechanisms including loss or inactivation of phosphatase and tensin homolog on chromosome 10 (PTEN), a tumor suppressor, and upregulation of tyrosine kinase growth factor receptors (RTKs) and oncogenes such as *ras* [6,7,26,27,28,29]. Recently, activation of PIK3CB gene encoding catalytic subunit p110β of PI3Kβ appears to be involved in the development of prostate and breast cancer [30,31]. Studies also indicate p85 regulatory isoforms also contribute to tumorigenesis by different mechanisms. Alterations in PIK3R1 and PIK3R2 genes encoding p85α and p85β, respectively, are reported to be associated with tumorigenic activity [32,33,34]. Due to these alterations in class I PI3Ks, the PI3K pathway is overactivated in many malignancies leading to activation of Akt/PIKB and downstream effector molecules promoting competitive growth and survival advantage, metastatic ability, and resistance to drug therapy. More than 50% of breast, prostate, ovarian, glioblastoma multiforme, uterine, and lung cancer cases are linked to the deregulation of the PI3K pathway [35,36,37].

### 2.2. Activation of Phosphatidylinositol 3-Kinase Signaling

**Figure 1:**
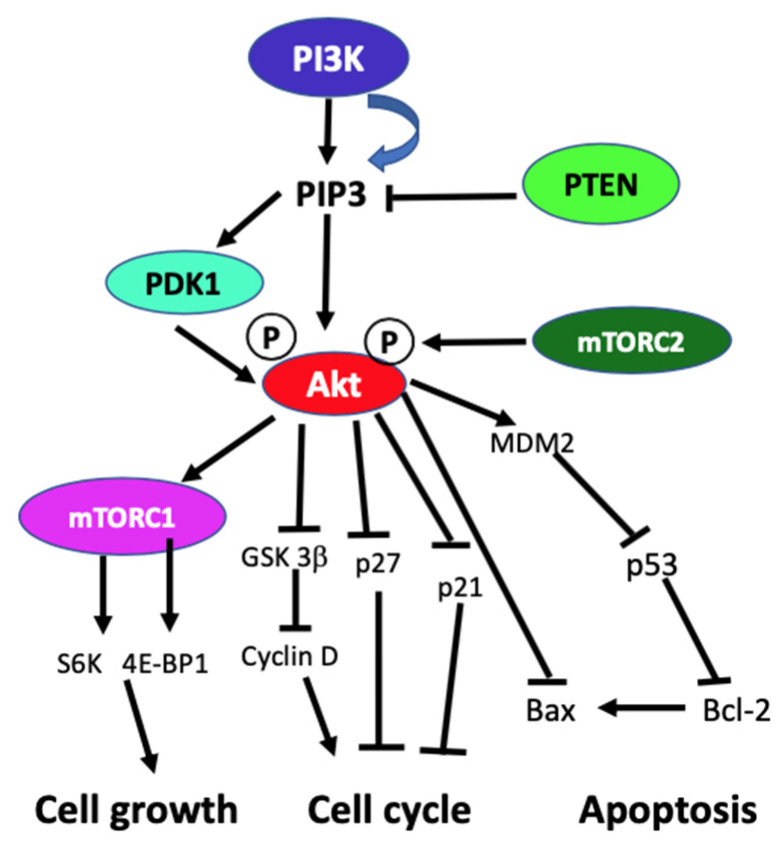
Schematic diagram of generic PI3K signaling displaying the signaling pathways of proteins leading to different cellular effects. In the figure, arrows indicate activation, and blunt end lines indicate inhibition.

In the absence of activating signals, the kinase activity of the p110 catalytic subunit of class IA PI3K is inhibited by its dimerization with the regulatory p85 subunit. Upon RTK or G-protein coupled receptor (GPCR) activation in response to extracellular signals, class IA p110/p85 heterodimer is recruited to the plasma membrane, where p85 separates from p110 catalytic subunit activating p110 kinase activity [38,39]. Activated p110 phosphorylates phosphatidylinositol 4,5-biphosphate (PIP_2_) to produce lipid second messenger phosphatidylinositol 3,4,5-triphosphate (PIP_3_). The PIP_3_ then binds and recruits a subset of pleckstrin-homology (PH), FYVE, Phox (PX), C1, C2, or other lipid domains of downstream targets to the cell surface membrane. Among these downstream targets, Akt and PDKI are the PH domain-containing proteins that are crucial for activating cell growth and survival pathways. Akt, one of the PI3K effector residing in the cytoplasm in an inactive form, translocates to the plasma membrane upon receptor activation of PI3K and binds to PIP3. Similarly, PDK1, also containing PH domain, translocates to the plasma membrane and binds to PIP_3_ upon PI3K activation. Upon translocation to the cell surface membrane, PDK1 partially activates Akt by phosphorylating Thr307. Full activation of Akt is achieved through phosphorylation of Ser473 by mTOR complex 2 (mTORC2), DNA-dependent kinase, or PDK2. Akt acts downstream to PI3K controlling multiple pathways that regulate cell proliferation, growth, survival, death, and metabolism through its downstream targets [1,2,3,40]. The other essential downstream target of PI3K is mTOR, which regulates protein synthesis essential for cell growth, proliferation, angiogenesis, and other cellular activities. It acts both downstream and upstream of Akt and it is active in two different multiprotein complexes, the target of rapamycin complex 1 (TORC1) and 2 (TORC2). The central role of PIP_3_ formed by the class I PI3Ks in all these cellular processes is negatively regulated by PTEN. PTEN downregulates class I PI3K activated pathways and PIP_3_ levels by converting PIP_3_ to PIP_2_ (Figure 1) [1,2,3,8,40,41].

### 2.3. Phosphatidylinositol 3-Kinase Inhibitors

PI3KIs are subdivided into pan-PI3K inhibitors, isoform-specific PI3K inhibitors, dual PI3K/mTOR inhibitors, mTOR, and Akt inhibitors [8,9,42]. Many of the PI3KIs are being used in preclinical and clinical trials to treat cancers. The pan-PI3KIs such as pictilisib (Taselisib, GDC-0941), apitolisib (GDC-0980), and buparlisb (BKM120) that target all class I isoforms exhibit similar efficacy. Since PIK3CA is frequently mutated in solid tumors, isoform-specific PI3KIs such as alpelisib (BYL719) and taselisib (GDC0032) that selectively target the PIK3CA protein product p100α are under preclinical and clinical testing. On the other hand, B cell signaling is upregulated in chronic lymphocytic leukemia through the hyperactivation of the PI3K pathway due to increased expression of PIK3CD protein product p110δ in hematopoietic cells and lymphoid tissue. Idelalisib, a specific inhibitor for p110δ, is the first PI3KI approved for the treatment of relapsed chronic lymphocytic leukemia in combination with CD-20 antibody rituximab [37]. However, clinical trials with early versions of PI3KIs used as single-target drugs in monotherapy have limited efficacy potentially due to compensatory or concurrent activation of other survival and growth-related signaling pathways [8,9]. Different and alternative strategies are being developed to block tumor growth and progression to overcome the limitations of a single target approach. Among these, the latest polypharmacology approach that is designed to overcome the limitations of single-target therapy appears to have a superior therapeutic effect along with reduced adverse reactions and diminished potential drug resistance. Specifically, the epigenetic polypharmacology approach which uses a dual-acting HDAC and PI3K inhibition by incorporating HDAC inhibitor functionality into a PI3K inhibitor pharmacophore is capable of potent anticancer activity by disrupting the oncogenic signaling network through simultaneous inhibition of HDACs and PI3K in cancer cells [15,16].

## 3. Histone Deacetylases (HDACs)

HDACs are a family of enzymes that play crucial roles in a number of biological processes essentially through silencing gene transcription. Gene expression is regulated by the contrasting action of HDACs and histone acetyltransferases (HATs). The HATs mediate the acetylation of histones promoting gene transcription, whereas HDACs suppress gene expression by deacetylating histones. Besides histones, functions of several nonhistone proteins that are essential for cell proliferation, differentiation, and apoptosis are regulated by acetylation/deacetylation.

### 3.1. Role of HDACs in Epigenetic Alterations

Apart from genetic mutations, alterations in epigenetic regulations are the main trigger for cancer initiation, progression, and metastasis [11]. Chromatin is a dynamic structure subject to remodeling through reversible epigenetic alterations in response to environmental *cues*. The architecture of chromatin is subject to dynamic remodeling from a highly condensed transcriptionally repressive state to a more relaxed transcriptionally permissible state in response to external and internal *cues*. Thus, permitting accessibility of DNA to the transcriptional machinery. Such chromatin remodeling is facilitated by epigenetic factors through various covalent posttranslational modifications (PTMs) to the N-terminal histone tails, including acetylation, methylation, phosphorylation, ubiquitination, and ADP-ribosylation. Among these epigenetic histone modifications, acetylation of histones plays a crucial role in the epigenetic regulation of gene expression. Histone acetyltransferases (HATs) and histone deacetylases (HDACs) represent two enzyme families that are crucial to epigenetic regulation of gene expression. Acetylation of lysine ε- amino group of lysine side-chain in the N-terminal tails of core histones (H2A, H2B, H3, and H4) is a reversible dynamic PTM regulated by contrasting activities of HATs and HDACs [10,11,12,13]. Transfer of acetyl group to the lysine residues of histones by HATs neutralizes their positive charges causing decondensed chromatin structure that is accessible to transcriptional activation [43]. Additionally, multiple specific sites are acetylated on core histones. These sites are recognized by bromodomain components found in certain proteins that are part of the chromatin-remodeling coactivator complexes involved in transcriptional activation [44]. In contrast, HDACs stimulate condensed and transcriptionally repressed chromatin structure by deacetylating histones and are associated with corepressor complexes that takes part in transcriptional suppression [10,11,12,13,45,46,47,48,49]. Besides acetylation, the lysine side chain of histones is a target of other PTMs such as methylation, sumoylation, and ubiquitination [50,51,52,53,54,55,56,57]. However, these PTMs are mutually exclusive on the same lysine residue, promoting a great potential for cross-regulation. Crosstalk between these different PTMs at independent sites of histone forms a ”histone code” that is translated to a specific biological outcome crucial to normal development, and disease pathogenesis [10,12,51,52,53,54,55,56,57].

Acetylation of lysine is a major PTM modification of histones and crossregulation between acetylation and another PTM is crucial in modulating chromatin-based transcriptional control [50,51,52,53,54,55,56,57]. It is now recognized that thousands of human proteins are subject to reversible acetylation and deacetylation [47,51,58]. Enrichment of acetylated proteins by immunoprecipitation with anti-acetyl-lysine antibodies, combined with high-resolution mass spectrometry-based proteomics, reveals lysine acetylation is a widespread and important epigenetic PTM that affects histones and some nuclear proteins, along with thousands of other human proteins [59,60]. Some of the acetylated nonhistone proteins include transcription factors, various other nuclear regulators, and cytoplasmic proteins, suggesting that lysine acetylation is not only crucial to the nuclear functions, but also essential for regulating various cytoplasmic processes, including cytoskeletal dynamics, energy metabolism, endocytosis, and signal transduction [47,51,52,61]. Moreover, several acetylated nonhistone proteins are key factors in different signaling pathways. In some nonhistone proteins, lysine acetylation occurs at multiple sites and crosstalk with phosphorylation, methylation, ubiquitination, and other PTMs, leading to a code-like multisite modification program that is crucial for the control of cellular signaling specific to a particular scenario. Considering that histones are not the only proteins that are subject to reversible acetylation and deacetylation, it is conceivable that HATs, HDACs, and bromodomains, the epigenetic players that add, erase, and read the acetyl group, respectively, have a broader impact on signaling pathways. Accordingly, different HATs and HDACs are now referred to as histone/lysine acetyltransferases (HATs/KATs) and histone deacetylase/lysine deacetylases (HDACs/KDACs), respectively. These enzymes play a critical role in nonhistone protein acetylation regulating signaling events essential for a range of cellular processes and physiological and pathophysiological functions [62,63,64]. Epigenetic-driven alterations in lysine acetylation due to dysregulated activities of several HATs/KATs, HDACs/KDACs, and bromodomain-containing proteins have been linked to various diseases, notably in cancer, but also in cardiovascular disease, neurological and immunological disorders [38,39,40,41,60,64,65,66,67]. A variety of HATs/KATs, HDACs/KDACs, and bromodomain inhibitors have been developed, studied, and used as anticancer agents in clinical trials.

### 3.2. Types of HDACs

Enzymatic deacetylation was first detected in 1977 when Friend erythroleukemic cells (FEC) were treated with n-butyrate [68]. Butyrate stimulated differentiation of FEC into hemoglobin-synthesizing normal-like cells. This phenotypic change was linked to histone hyperacetylation, indicating the presence of HDAC, and butyrate is recognized as HDACI. There are 18 different mammalian HDACs that have been identified. HDACs are classified into four classes I, II, III, and IV [6,10,12,45]. Class I, II, and IV are referred to as classical HDACs and class III enzymes are known as sirtuins. Class I, II, and IV HDACs are dependent on Zn^2+^ for the deacetylase activity. Class I HDACs include HDAC1, HDAC2, HDAC3, and HDAC8 that have sequence similarity to yeast counterpart RPD3. Class II HDACs are related to yeast enzyme HDAI and are divided into subclasses IIa and IIb. The IIa includes HDAC4, HDAC5 HDAC7, and HDAC9, whereas IIb includes HDAC6 and HDAC10. Class IV consists of only HDAC11. Class III includes seven NAD^+^-dependent sirtuins that are related to yeast counterpart Sir2.

HDACs have an important role in cytoplasmic functions besides their epigenetic functions regulating gene expression. A number of cytoplasmic nonhistone proteins are acetylated by HDACs. These proteins include transcription factors, transcription regulators, DNA repair enzymes, chaperone proteins, signal transduction mediators, cytoskeletal proteins, and inflammation mediators [12,13,69,70,71]. Accordingly, histone deacetylases/lysine deacetylases (HDACs/KDACs) are directly or indirectly involved in several cellular functions such as gene expression, signaling pathways, protein stability, protein-protein interaction, eventually affecting cell proliferation, growth, differentiation, apoptosis, migration, and angiogenesis [12,13,69,70,71]. Moreover, altered expression of HDACs has been observed in many cancerous and noncancerous diseases, particularly in cancer, advocating HDACs are important therapeutic targets for anticancer therapies [72,73,74]. Therefore, the development of HDAC inhibitors is essential to the therapeutic intervention of epigenetic diseases, cancer in particular.

## 4. Histone Deacetylase Inhibitors (HDACIs)

The cancer cells harbor the epigenetic abnormalities required for oncogenesis by altering the epigenetic landscape of chromatin. Especially acetylation, one of the predominant modifications in epigenetics, serves as a key regulatory mechanism for gene expression. The global dysregulation of epigenetic histone acetylation modifications results in altered gene expression contributing to the establishment of the disease state. Aberrant epigenetic alterations are the important drivers in cancer pathogenesis often due to the silencing of tumor suppressor genes or overexpression of oncogenes. Moreover, the cancer cells with a unique portfolio of epigenetic changes are different from their normal counterparts. Accumulation of these altered epigenetic changes allows cancer cells to solidify their exclusive phenotype through dysregulated gene expression changes [75,76,77]. Furthermore, the accumulation of altered epigenetic changes can also promote resistance to therapy through the acquisition of pro-survival signaling [75,78]. The epigenetic histone acetylation alterations induced by aberrant expression of HDACs can be reversed by pharmacological inhibition of HDACs with HDACIs. Although HDACIs affect gene expression on a global level, their actions are specific to the malignant phenotype and have been recognized that only about 8–20% of genes are affected by HDACIs [79]. Additionally, HDACIs also indirectly affect gene expression by inhibiting HDACs interactions with nonhistones.

The HDACIs are a class of small-molecules that inhibit the activity of Zn^2+^-dependent classical HDACs (class I, II, and class IV HDACs) and promote acetylation of histone and nonhistone protein substrates. Originally, HDACIs were known for their varied cellular effects on cancer cells, such as inhibiting cell proliferation and stimulating differentiation or apoptosis, advocating their usefulness in anticancer drug discovery, and development [80,81]. Now, it is recognized HDACIs induce an array of cellular effects [69,82,83]. They stimulate cell cycle arrest by upregulating p21Cip1 and downregulating cyclins [69,84,85]. They also do the following: 1) Induce intrinsic and extrinsic apoptosis pathways causing apoptosis [75,86,87]. 2) Induce autophagy [88]. 3) Promote anti-angiogenic effect by altering Hif-1α function and downregulation of VEGF [89]. 4) Alter expression of nonhistone proteins including transcription factors and regulators, inflammation/immune response mediators, chaperones, DNA repair enzymes, and structural proteins [69,82,83,90]. These features of HDACIs are exploited as promising anticancer epi-drugs and several HDACIs have entered clinical trials in both hematologic and solid tumors [70,72,73,74,75,81,82,83,90]. As such, HDACIs are investigated mainly as anticancer epi-drugs, even though HDAC enzymes also play a crucial role in other diseases such as cardiovascular disease [91], neurological disorders [92], immunological processes and viral infections [93], and other disorders [94].

### 4.1. Types of Histone Deacetylase Inhibitors (HDACIs)

Several HDACIs have been either isolated from natural sources or synthesized with differing efficacy, potency, and pharmacokinetics [81,95]. The active site of Zn^2+^-dependent HDACs consists of a tubular pocket and a Zn^2+^ ion at the bottom of the pocket. The canonical pharmacophore of the HDACIs is composed of three different parts. A cap structure (CAP) that interacts with the rim of the tubular catalytic pocket of HDACs, a zinc ion binding group (ZBG), and a linker spanning the length of the tubular pocket responsible for connecting the cap and the ZBG [12,13,96,97]. Based on their Zn^2+^-binding structure, HDACIs are classified into different classes. These include hydroxamates, carboxylic acids, benzamides, and cyclic peptides, with differing potency, selectivity, and toxicity [48,69,98]. Hydroximic acid based HDACIs include trichostatin A (TSA), vorinostat (SAHA, suberoylanilide hydroxamic acid), panobinostat, belinostat, and several others. While TSA is used only in laboratory studies because of its toxicity, vorinostat, panobinostat, belinostat, and several others are approved for hematologic malignancies. Most of the hydroximic acid-based HDACIs are pan-HDACIs. The carboxylic acid type of HDACIs includes short-chain fatty acids such as butyric acid, phenylbutyrate, and valproic acid, which are weak inhibitors of HDAC class I and II. Butyrate and phenylbutyrate acids are in several phase II and phase I trials, respectively. Valproic acid is approved for epilepsy, bipolar disorders, and migraine. Among the benzamides, entinostat and chidamide (HBI-8000) are HDAC class I inhibitors. Entinostat is in phase II trials for several different hematologic and solid cancers. The cyclic tetrapeptides include the bicyclic depsipeptide romidepsin (FK228, FR901228). It is an HDAC class I inhibitor that has been approved for treating cutaneous T-cell lymphoma.

Most HDACIs are considered pan-inhibitors because they display a similar mechanism of action on different classical HDACs except for class IIa HDACs. HDACIs bind to the zinc atom in the catalytic pocket in a non-competitive manner but lacks selectivity. Thus, it is difficult to discern which classical HDAC enzyme inhibition is responsible for the therapeutic or toxic effects observed in clinical trials. To achieve desirable clinical outcomes, it would be appropriate to know which HDAC is contributing to the pathogenesis so that HDAC-specific inhibitors can be developed in the future.

### 4.2. Histone Deacetylase Inhibitors in Clinical Trials

The early drugs like butyrate targeting HDACs are used based on phenotypic effects in cancer models without knowing the specific targets. The observations that HDACIs exhibit varied cellular effects on cancer cells such as inhibiting cell proliferation and stimulating differentiation or apoptosis, point to their usefulness for anticancer drug discovery and development [80]. During the past ten plus years, several promising HDACIs have surfaced as effective anticancer epi-drugs. Because of the transient and reversible nature of epigenetic modifications and also based on their antiproliferative and proapoptotic effects in cancer cells, several HDACIs are approved for cancer treatment by the United States Food and Drug Administration (US FDA) [72,73,74,99]. Vorinostat, also (suberoylanilide hydroxamic acid, SAHA), a hydroxamic acid, is the first pan-HDACI approved in 2006 for the treatment of rare refractory cutaneous T-cell lymphoma (CTCL). Romidepsin (FK-288, FR901228, cyclic depsipeptide), a natural prodrug having inhibitory activity against class I HDACs, is approved in 2009 for the treatment of rare refractory CTCL. Belinostat (PXD101), and panobinostat (LBH589) are two other approved hydroxamate-containing pan-HDAC inhibitors. Belinostat is approved for the treatment of peripheral T-cell lymphoma in 2014 and panobinostat for the treatment of refractory multiple myeloma in 2015 [99]. Benzamide-based HDACI chidamide (HBI-8000) which inhibits HDAC1, 2, 3 and 10 has been approved for the treatment of refractory peripheral T-cell lymphoma in China [72,73,74,99]. Class I-selective benzamide containing entinostat and mocetinostat are in clinical trials for several solid tumors. Even though the HDAC-specific epi-drugs display potent anticancer activities on hematological malignancies, they exhibit limited effectiveness in supporting persistent suppression of solid tumors in monotherapies [100,101,102]. The single epi-drug therapeutic strategy is frequently ineffective in providing enduring tumor suppression. One of the main reasons for ineffectiveness is, cancer cells can evade drug-induced effects by eliciting other compensatory survival pathways due to the emergence of pharmacological resistance. Interestingly, HDACIs also exhibit anticancer effect by disrupting pro-survival signaling through epigenetic drug-induced sensitization mechanisms [75].

### 4.3. Disruption of Pro-Survival Signaling by HDACIs

Epigenetic inhibitors can disrupt pro-survival signaling in cancers that have developed resistance to therapy. During oncogenesis, epigenetic alterations can dysregulate the expression of certain proteins such as growth factor receptors or proteins associated with apoptosis that provide survival advantage causing resistance to therapy [75,103]. Increased expression of growth factor receptors in cancer cells can allow them to become resistant to therapy by over-activating the downstream pathways; for example, the PI3K/Akt pathway that leads to inhibition of cell death [104]. Despite the efforts to alleviate the effects of growth factor receptors by targeted therapies against it, such therapies are inadequate because of the rapid development of resistance. The use of HDACIs to control the expression of growth factor receptors appears to be a promising approach; accordingly, in breast cancer dacinostat, an HDACI disrupted EGF-mediated signaling that is linked to increased metastasis and cell survival [105]. This effect was achieved by downregulating the expression of*HER2* (human EGF-receptor-2) through two independent epigenetic mechanisms: by reducing *HER2* mRNA level, and by increasing proteasomal degradation due to dissociation from its chaperone protein HSP90 through enhanced acetylation. Additionally, a combination of HDACI entinostat and Her2/EGF-receptor kinase inhibitor, lapatinib synergistically inhibited Akt signaling to promote apoptosis in HER2-overexpressing breast cancer cells [106].

HDACIs can also disrupt pro-survival signaling by enhancing apoptosis signaling pathways. The binding of tumor necrosis factor-related apoptosis-inducing ligand (TRAIL) to its receptors death domain-containing receptor (DR) initiates pro-death signaling receptor expression stimulating apoptosis through caspase cascade [107]. Cancer cells are frequently resistant to apoptosis due to reduced DR receptor expression. These cells can be sensitized to overcome resistance to undergo cell death by treating them with HDACIs. Exposure of breast cancer cells to vorinostat sensitized the cancer cells to TRAIL-induced apoptosis by upregulating DR5 receptor expression [108,109].

Additionally, HDACIs can also sensitize cancer cells to overcome their dysregulated cell cycle control and diminished activities of DNA damage repair pathways through their ability to reverse dysregulated gene expression. Entinostat, an HDACI can sensitize breast cancer cells to doxorubicin-induced growth arrest by downregulating the expression of *myc,* E2F, and other G2M cell cycle genes. [75]. Entinostat in combination with decitabine, a DNA methyltransferase inhibitor, is able to restore cell cycle control in pancreatic cancer through upregulation of p21 [75].

### 4.4. Mechanisms of Resistance to HDACIs

Cancer cells are highly resilient and adaptable in their ability to respond and to evade growth inhibitory and toxic factors to survive and resist death. They constantly adjusting to overcome the effects of damaging agents and environmental constraints to exist independently free from the influence of external proliferative/survival stimuli. These acquired stable alterations may hinder cancer cells response to the effects of HDACIs through developing different mechanisms to survive and achieve resistance. A thorough understanding of molecular determinants of resistance to HDACIs may provide the basis for therapeutic options with enhanced efficacy. Resistance to HDACIs is often seen and involves different mechanisms [75,110,111]. Some of the resistance mechanisms to HDACIs involves cell cycle proteins, thioredoxin expression, apoptosis-related proteins, signaling proteins, and NF-kB expression.

Cell cycle proteins: It has been implicated that the induction of p21Cip1 in response to HDACIs is responsible for cell cycle arrest and serves to play a protective role. Several studies have shown that upregulated expression of p21Cip1 in response to HDACIs mediates cell cycle arrest and differentiation or apoptosis [112]. When p21-deficient HCT116 cells are treated with romidepsin, cells get arrested in the G2 phase compared to wild type cells [113]. The p21 is considered as a negative regulator of the cell cycle and a cyclin-dependent kinase inhibitor implicated in apoptosis [114]. Studies in U937 leukemia cells demonstrated that HDACI-induced p21Cip1 protected the cells from apoptosis, and blocking its expression resulted in apoptosis [115].

Thioredoxin expression: Resistance to HDACIs can be linked to an increased capacity of cancer cells to resist oxidative stress. HDACIs increase ROS production and appear to be an important factor in the proapoptotic effects of HDACIs. Increased expression of thioredoxins, peroxiredoxins, and redox proteins that protect cells from ROS may acquire resistance to HDACIs. Treatment of cancer cells with HDACIs causes oxidative stress by increasing ROS levels [116]. Cell death induced by HDACIs can be partially rescued by preincubating with free radical scavenging N-acetylcysteine.

Apoptosis-related proteins: Increased expression of antiapoptotic proteins has been shown to prevent HDACIs-mediated cell death. Increased levels of Bcl-2 and Bcl-x cause resistance to the treatment of vorinostat, dacinostat, panobinostat, or oxamflatin [117,118]. The knockdown of proapoptotic Bax in panobinostat-sensitive T-cell lymphoma cell lines resulted in diminished toxicity [119]. On the other hand, knockdown of antiapoptotic Mcl-1 enhances HDACI-mediated apoptosis in primary chronic lymphocytic leukemia cells and K562 cells [120]. These reports indicate silencing of proapoptotic proteins blunts HDACIs-induced cell death.

Signaling proteins: Resistance to HDACIs is significantly associated with activation of PI3K and MAPK pathways. A combination of panobinostat with compounds that abolish PI3K and MAPK signaling result in synergistic cytotoxicity may be due to increased ROS [121]. A combination of romidepsin with inhibitors of Akt resulted in synergistic cytotoxicity indicating phosphorylated Akt is an important resistance mechanism to romidepsin. These and other combination studies targeting different signaling molecules appear to cause resistance. Signal transducer and activator of transcription (STAT) pathway also contributes to resistance to HDACIs. In a group of almost 40 lymphoma cell lines, activation and expression of STAT-1, -3 and -5 was higher in cell lines that were more resistant to vorinostat than in sensitive cell lines [122]. Analysis of a series of skin biopsy for the nuclear staining of phosphorylated STAT3 recognized that patients with strong staining were more likely resistant to vorinostat resistance [122]. The combination treatment with vorinostat and compounds that target phosphorylated STAT3 may increase the efficacy.

NF-kB activation: The activation of the NF-kB pathway occurs in a number of cancers and leads to the deactivation of the apoptotic pathway and increased cell survival [123]. It has been identified as a mediator of resistance to HDACI treatment. Inhibition of HDAC by HDACIs enhances transcriptional activation of NF-kB through acetylation of RelA/p65 subunit leading to the induction of an array of genes involved in protection against cell death [124]. The activation of NF-kB by HDACIs interferes with the triggering of cell death. In non-small lung cancer cell lines and leukemia cells, activation of NF-kB by HDACIs triggers cell death [125,126]. Inhibition of NF-kB by its inhibitor Bay-11-7085 sensitizes the cancer cells to death in response to inhibition of HDAC.

Additionally, HDACIs can also sensitize cancer cells to overcome their dysregulated cell cycle control and diminished activities of DNA damage repair pathways through their ability to reverse dysregulated gene expression. Entinostat, an HDACI can sensitize breast cancer cells to doxorubicin-induced growth arrest by downregulating the expression of *myc,* E2F and other G2M cell cycle genes. [75]. Entinostat in combination with decitabine, a DNA methyltransferase inhibitor, is able to restore cell cycle control in pancreatic cancer through upregulation of p21 [75].

## 5. Therapeutic Strategies

In cancer, genetic, epigenetic, and metabolic factors all participate in neoplasia by altering the molecular networks that control cell proliferation, growth, differentiation, migration, and cell death. Deregulation/misregulation of different molecular networks and misunderstanding in cross-talks between epigenetic and non-epigenetic players that are linked to various cellular processes play important role in the etiology of cancer. Supporting these effects, the aberrant expressions of HDACs promote altered epigenetic modifications that endorse abnormal gene expression profiles [72,73,74,127]. The knockdown of several HDACs in some of these cancer cells stimulated cell cycle arrest and apoptosis supporting the aberrant expression of HDAC activity is linked to cancer [97]. Relevantly, HDACIs have a range of anticancer activities through the collaboration of their primary chromatin-associated effects on gene transcription and their cellular effects [68,128]. These features of HDACIs supports targeting epigenetic aberrations to prevent carcinogenesis, and open opportunities for HDACIs as the class of anticancer agents. Several of these HDACIs have already been approved by the US FDA for cancer treatment [see Section 4.1] and are in preclinical and clinical testing.

Although the HDACIs, when used as single agents, display potent anticancer activities on hematological malignancies, they exhibit limited effectiveness in the suppression of solid tumors in monotherapies [101,129]. The single HDAC therapeutic approach is frequently ineffective in providing sustained tumor suppression because cancer cells can dodge drug-induced effect due to pharmacological resistance, and also because of the toxicity and off-target effects of epi-drugs [130,131,132,133]. This letdown has encouraged new drug design and therapeutic approaches to provide lasting tumor suppression. The goal of drug discovery in the epi-drug research is no longer just the design of highly potent and selective drugs acting on a specific epi-target, but advancing towards designing network-active compounds with multitargeting capability through a polypharmacology approach [14,15,16,131,132,133,134].

### 5.1. Alternate Strategies Using Network-Active Compounds

The objective of the polypharmacology approach is to simultaneously hit all different targets linked to the onset and development of a particularly complex disease at different levels so that improved therapeutic effect is achieved [14,15,16,131,132,133,134]. The goal is to achieve a synergistic effect by hitting a cellular pathway at different levels by using multitarget drugs. Synergistic therapeutic effects can be achieved by three alternative strategies even though they exhibit striking differences. These include multi-medication therapy (MMT, drug combination), multi-compound medication (MCM, co-formulation of multiple active compounds), and multitarget-directed ligands (MTDLs, single multitargeting compounds) [15,16]. Both MMT and MCM strategies have their own specific targets but are based on the combination or association of two or more active principles, whereas MTDLs strategy is designed to create a single molecule with an ability to simultaneously interact with different targets. Specifically, a combination of epi-drugs with other anticancer agents including kinase inhibitors, chemotherapeutic agents, hormonal inhibitors, and other epi-drugs for which a rationale has been defined to benefit from synergistic effects of drug combinations have been designed [15,16,131,132,133,134]. Some of them are under preclinical and clinical testing. Importantly, among the several hybrid drugs produced, CUDC-907 is a promising orally bioavailable small-molecule dual HDAC-PI3K inhibitor [15,16,134,135,136,137,138,139,140,141,142,143,144]. CUDC-907 simultaneously inhibits HDACs and PI3K causing a synergistic effect. It is designed by introducing hydroxamic acid moiety as the zinc binding functional group to a morpholinothienopyrimidine-based PI3K pharmacophore through an appropriate linker [135]. Recently, another dual HDAC-PI3K hybrid drug is reported, which is designed by introducing hydroxamic acid moiety as the zinc binding functional group to a quinazoline-based PI3K pharmacophore through an applicable linker [143]. Different therapeutic approaches involving HDAC-specific epi-drugs and PI3KIs directed against neoplastic diseases are narrated in the following section.

#### 5.1.1. HDACs-Specific Epi-Drugs Targeting HDACs

HDACIs induce multiple cellular effects specific to cell types through the mobilization of various molecular pathways. Among these, the PI3K pathway is the most prominent pathway affected by HDACIs. Since, dysregulation of PI3Ks and their downstream effector molecules forming the PI3K/Akt/mTOR axis contribute to cancer initiation, progression, and growth, HDACIs are used as single drugs to test whether their anticancer effect involves inhibition of PI3Ks in different cancer cells and in animal xenograft cancer models. Moreover, determining the efficacy of single drugs is a prerequisite for designing a multidrug combinational approach.

Sodium butyrate, an HDACI, inhibits cell proliferation, induces differentiation, or promotes apoptosis in a variety of tumor cells. Gastric BGC823 cells treated with butyrate resulted in inhibition of cell proliferation, altered cellular morphology, and increased expression of PTEN and mucosal factor MUC2, but decreased expression of PI3K [145]. These effects are heightened by intervention with PI3K inhibitors and lessened with PTEN siRNA indicating sodium butyrate induced upregulation of PTEN and MUC2 expression and differentiation of gastric cancer cells are mediated through the PTEN/PI3K signaling pathway [145].

**Figure 2:**
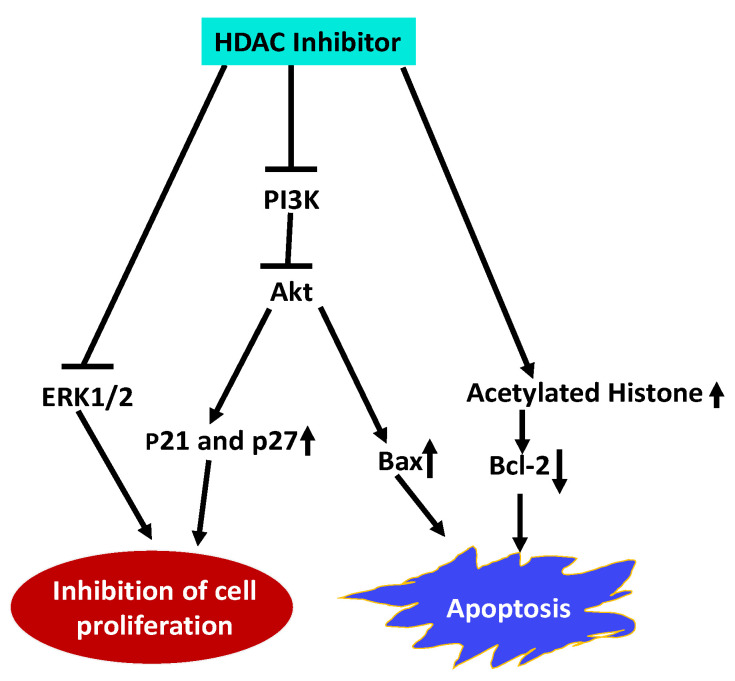
Schematic representation of HDACI Trichostatin A (TSA) effects on Esophageal squamous cell carcinoma (ESCC). TSA inhibits ESCC proliferation, and arrests cells in G1 phase by upregulating by p21Cip1 and p27Kip1. It inhibits cell proliferation by inhibiting PI3K expression, and reducing activation of Akt and ERK1/2. Promotes apoptosis by altering expression of pro- and anti-apoptotic proteins, and increases histone acetylation [146]. (Blunt end lines indicate inhibition, upward arrows indicate upregulation and downward arrows indicate downregulation of proteins).

Esophageal squamous cell carcinoma (ESCC) is a highly malignant and lethal disease. Treatment of ESCC cells with Trichostatin A (TSA), an HDACI, inhibited proliferation of ESCC cells and arrested the cells in the G1 phase by upregulating p21Cip1 and p27Kip1 cell cycle inhibitors [146]. TSA also induced apoptosis by upregulating pro-apoptotic protein Bax and downregulating anti-apoptotic Bcl-2. Furthermore, TSA inhibited the expression of PI3K and reduced the phosphorylation of Akt and ERK1/2 along with an increase in acetylated histone H4. These results indicate that TSA impedes ESCC cell proliferation and promote apoptosis by inhibiting HDAC activity and activation of PI3k/Akt and ERK1/2 pathways and (Figure 2).

Panobinostat, approved for the treatment of multiple myeloma patients, was tested for its efficacy on NB4 cells and primary acute promyelocytic leukemia (APL) patient cells. The results indicate panobinostat not only effectively reduces the survival rate of both NB4 and APL primary cells, but also eliminated the stimulatory survival effect of microenvironment signals transduced by mesenchymal stem cells (MSC) on APL cells by incorporating PI3K inhibitor when APL cells are co-cultured with MSC [147].

Romidepsin (depsipeptide, FK228) approved for the treatment of cutaneous and peripheral T-cell lymphoma treatment directly inhibits PI3K activity and strongly promotes apoptosis through its HDAC/PI3K dual inhibition [148]. Furthermore, FK-A11, an analog of depsipeptide, is reported to be the most potent HDAC/PI3K dual inhibitor exhibiting antitumor activity in HT1080 fibrosarcoma and PC3 prostate cancer cell xenograft mouse models [149].

Vorinostat (SAHA, Zolinza) is a good efficacious, well-tolerated drug that has been used in the treatment against CTCL. It also exhibits anti-solid tumor effects for other types of cancer in monotherapy and also in combination therapy. To understand the mechanism of Vorinostat anti-solid tumor activity, its effects on cervical cancer were investigated [150]. The outcome of these studies revealed that Vorinostat inhibits cell proliferation, migration, and invasion of cervical cancer cells, arrest cells in S phase, and induces apoptosis. Furthermore, Vorinostat inhibited PI3K (p110α), p-PI3K p55, and p-Akt protein expression and upregulated major histocompatibility class I-related chain A (MICA) expression in vitro and in vivo, which promoted natural killer (NK) cell-mediated cervical cancer cell lysis. These studies indicate that vorinostat exhibits anti-solid tumor activity by upregulating MICA expression in cervical cancer cells through the mediation of the PI3K/Akt pathway, which enhances the susceptibility of cervical cancer cells for NK cell-mediated cytolysis.

#### 5.1.2. Multi-Drug Combination Targeting HDACs Activity and PI3K Pathway

Simultaneous use of two or more single-target inhibitors that are previously validated as effective in monotherapy can be used for combination therapy to stimulate a synergistic effect. Blocking multiple signaling nodes arising from HDACs and PI3K pathways simultaneously with HDACI and PI3KI can overcome the inadequacies of single therapeutic agents. A combination of HDACI/PI3KI, and HDACI/dual PI3K-mTOR inhibitors are currently widely used in preclinical studies and in the clinics for the treatment of both hematologic and solid malignancy [15,16,133].

**Figure 3:**
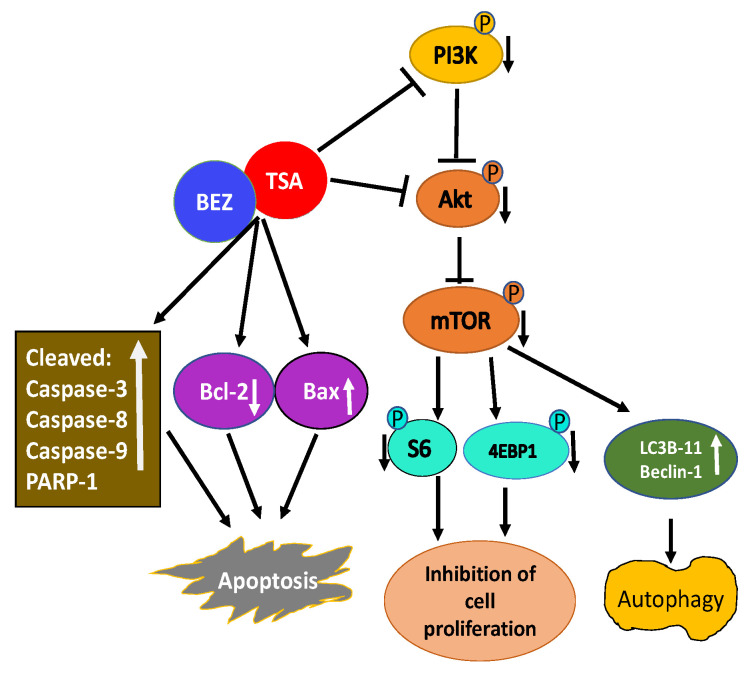
Schematic illustration of activity of TSA/BEZ235 combination on breast cancer cells. Combination treatment of TSA/BEZ235 results in synergistic suppression of breast cancer cells [152]. Through reduced activation of PI3K/Akt/mTOR and their downstream targets, TSA/BEZ235 drug combination accounts for inhibition of cell proliferation and apoptosis. The anti-breast cancer effect of drug combination is through both the mitochondrial and the death receptor pathway. TSA/BEZ235 combination also causes autophagy. (blunt end lines indicate inhibition, upward arrows indicate upregulation and downward arrows indicate downregulation of proteins).

A combination of TSA and PI3K-mTOR dual inhibitor BEZ235 was used to test their effectiveness on breast cancer cells [151]. Their combination synergistically inhibited the growth of multiple breast cancer cell lines. Mechanistic studies revealed a combination of TSA and BEZ235 reduced phosphorylation of downstream targets of PI3K including Akt, mTOR, S6, and 4EBP-1 accounting for inhibition of cell proliferation and apoptosis. Further analysis revealed an increase in cleaved caspase-3, caspase-8, caspase-9, and poly-ADP ribose polymerase-1 (PARP-1), along with the reduced expression of Bcl-2 and increased expression of Bax indicating the combination of TSA and BEZ235 causes an anti-breast cancer effect through both mitochondrial pathway and the death receptor pathway (Figure 3). Co-treatment of breast cancer cells with TSA and BEZ235 also caused autophagy based on LC3B-II and Beclin-1 increased levels. In-vivo studies further revealed a combination of TSA and BEZ235 blocked tumor growth without any noticeable side effects. Taken together, TSA and BEZ235 drug combination approach has significant application in the treatment of breast cancer patients [151].

Recent evidence indicates frequent mutation in histone modifying proteins and dysregulation of PI3K pathway in non-Hodgkin lymphoma (NHL), particularly in diffuse large B-cell lymphoma (DLBCL). This observation prompted exploration of the effectiveness and an assessment of the mechanisms of action of co-administration of panobinostat and BEZ235 in DLBCL cells [152]. Panobinostat and BEZ235 interact synergistically in ABC-, GC-, and double-hit DLBCL, but not in normal CD34^+^ cells. The synergistic effect of panobinostat and BEZ325 was displayed by significant Akt dephosphorylation and GSK3 dephosphorylation/activation. Drug combination promoted apoptosis as revealed by abrogation of p21Cip1 induction, Mcl-1 downregulation, Bim upregulation, and increased Bcl2/Bcl-xl binding and reduced Bax/Bak binding to Bcl/Bcl-xl/Mcl-1. Additionally, co-administration of panobinostat and BEZ325 increased H2A.X phosphorylation and histoneH3/H4 acetylation. These effects were collectively linked to the synergism of panobinostat and BEZ235. The synergistic effect of a combination of panobinostat and BEZ235 underscores the therapeutic potential of a drug combinational approach.

Sodium butyrate exhibited inhibitory effects on an aggressive metastatic human colon cancer cell line, KM20 [153]. Sodium butyrate-mediated effect on these cells was enhanced by the inhibition of the PI3K pathway resulting in apoptosis and reduced viability of KM20 cells. Co-incubation of KM20 cells with butyrate and PI3K inhibitor LY294002 or wortmannin enhanced colon cancer cell apoptosis by activating caspase 9 and caspase 3. This finding indicates the anticancer effect of HDACI can be synergized with a PI3KI, and effective in colon cancer treatment.

In tumorigenic non-small cell lung cancer, (NSCLC) resistance to HDACIs is mediated through the activation of nuclear factor-kB (NF-kB) via PI3K/Akt-dependent pathway. Whether inhibition of the PI3K/Akt pathway will allow the NSCLC cells to overcome the resistance to HDACI-induced apoptosis was investigated using butyrate [154]. Treatment of NSCLC cell lines with butyrate activated NF-kB-dependent transcription and this effect was inhibited by LY294002, a PI3KI. Combined treatment of NSCLC with butyrate and LY294002 increased apoptosis by increasing caspase-3 level and DNA fragmentation. In vivo, combined treatment of butyrate and LY294002 was tumoristatic with decreased tumor growth and increased apoptosis. Decreased phospho-Akt level and increased acetylated histone H3 level in tumor tissues treated with butyrate and LY294002 indicating NSCLC xenografts can be sensitized to undergo apoptosis with combined treatment with HDACI and PI3K/Akt pathway inhibitors.

Another study supports the superior efficiency of HDACI and BEZ235 combination treatment against NSCLC [155]. Heightened activities of HDACs and PI3K/Akt signaling pathways have been associated with NSCLC development and progression. Co-treatment of NSCLC cells with TSA and BEZ235 reveals a synergistic effect on the inhibition of NSCLC cell proliferation and stimulation of apoptosis. This combination also synergistically inhibited NSCLC migration, invasion, and the NSCLC epithelial-mesenchymal transition in vitro. Furthermore, reduction in xenograft growth and metastasis rates and ki-67 protein expression in vivo in response to co-treatment with TSA and BEZ235 also provides evidence for synergistic efficacy of the co-treatment, warranting further evaluation of combination treatment approach for NSCLC.

Head and neck squamous cell carcinoma (HNSCC) is the sixth most common cancer in the developed world. Despite improved surgical and radiological treatment of HNSCC, it continues to be a serious disease and needs new treatment approaches. This situation brings HDACIs into the picture for the treatment option. Studies have revealed that a variety of HDACIs exhibit anticancer effects on squamous cell carcinoma (SCC) in vitro and in recent patient trials. However, the patient trials have shown that HDACIs have limited therapeutic potential in monotherapies for the treatment of HNSCC [156]. Since it is possible to boost anticancer effects of HDACIs by selective targeting of the PI3K/Akt pathway, the effect of PI3K, Akt, and dual PI3K/mTOR inhibitors either alone or in combination on the therapeutic potential of HDACIs in HNSCC cell lines and xenograft HNSCC models was investigated [157]. Treatment of HNSCC cell lines with PI3K, Akt, and dual PI3K/mTOR inhibitors demonstrate increased in vitro cytotoxicity induced by HDACIs. Furthermore, HDACIs and PI3KIs inhibited tumor growth of HNSCC xenograft models. In agreement with this effect, intra-tumoral HDAC and PI3K inhibition was observed as evaluated by histone H3 acetylation status and phospho-Akt staining, respectively.

Glioblastoma multiforme (GBM) is a common malignant brain tumor. GBM cells treated with Panobinostat and BEZ235 alone or in combination were assessed for cell viability, proliferation, and apoptosis, and mechanisms of cytotoxicity were evaluated [158]. The combination approach with HDACI and PI3KI synergistically inhibited cell viability, significantly inhibited cell proliferation, and stimulated apoptosis. Additionally, co-treatment also increased caspase 3/7 activity, suppressed markers of cell proliferation and anti-apoptotic markers, and Akt signaling. These effects of a combination approach with HDACI and PI3K/mTOR inhibitor are promising and necessitate further evaluation in GBM therapy.

Medulloblastoma (MB) is a common malignant pediatric brain tumor. Based on genetic studies there are four different molecular groups of MB. The most aggressive MB among these groups exhibits amplification or overexpression of *myc* oncogene. Patients with *myc*-driven MB reveal poor prognosis and need more effective treatment. Using an animal model of *myc*-driven MB, the effectiveness of HDACIs was tested for their ability to decrease the viability of tumor cells [159]. HDACIs not only potently inhibit the survival of *myc*-driven MB cells in vitro partly due to the upregulation of the FOXO1 tumor suppressor gene, but also synergize inhibition of tumor growth in vivo in combination with PI3KIs. These effects recognize the effectiveness of combination therapy for the most aggressive form of MB.

#### 5.1.3. Polypharmacology-Based Approach Targeting HDAC Activity and PI3K Pathway

**Figure 4:**
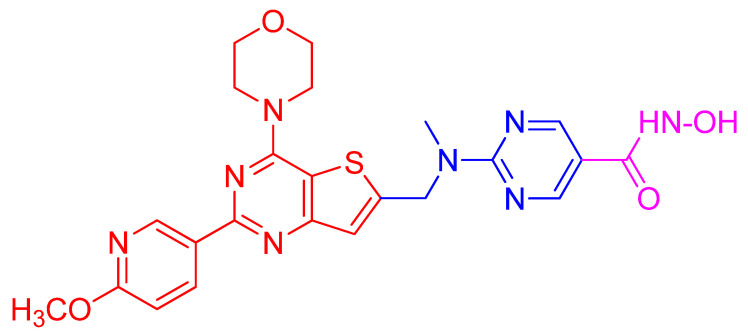
Structure of the HDAC/PI3K hybrid inhibitor CUDC-907. Hydroxamic acid moiety as the zinc binding functional group of HDACI (shown in magenta) is conjugated to morpholino- pyrimidine PI3K pharmacophore (shown in red) through a linker (shown in blue).

While the traditional multi-drug combination therapy approach is based on a combination of two or more drugs, each with its own specific target, the polypharmacology approach is designed to develop a single-hybrid drug to simultaneously interact with multitargets that are responsible for the onset of a disease. The hybrid drugs are created by conjugating two or more active molecules that individually exhibit a confirmed activity against specific targets. Such a multitarget hybrid drug strategy has been used to develop a dual HDAC-PI3K inhibitor hybrid drug that simultaneously inhibits HDACs and PI3K pathway in cancer cells. Prior to developing the multitarget hybrid drug, to confirm the potential synergistic response of HDAC and PI3K inhibition, individual effects of HDACI vorinostat (SAHA) and PI3K inhibitor pictilisib (GDC-0941) on growth inhibition of human PC-3 prostate cancer cells were determined [135]. Then the combined effect of vorinostat and pictilisib was compared to their individual effect and analyzed using the median effect analysis [160]. The analysis indicated significant combination index that was less than 1, which provided a rationale for the development of a single dual HDAC and PI3KI hybrid drug. Based on this information, multitarget inhibitors were designed and synthesized by integrating HDAC inhibitory hydroxamic acid functionality (vorinostat, panobinostat, JNJ-16241199) into a core structure of morpholinothienopyrimidine pharmacophore that is shared by several PI3KIs such as apitolisib (GDC-0980), pictilisib (GDC-0941), PI-103 and BKM120 (Buparlisib [135]. One of these hybrid drugs identified is CUDC-907 (Figure 4). It was tested to confirm whether it displays potent pan-inhibitor activity against HDAC class I and II enzymes, and pan-inhibition against class I PI3Ks. Its potency against class I HDAC was found to be like that of panobinostat and greater than vorinostat [135]. Similarly, CUDC-907 revealed potent inhibition of class I PI3Ks with an IC_50_ of 19, 54, and 39 nmol/L for PI3Kα, P13Kβ, and PI3Kδ, respectively. This activity is comparable to that of known PI3KI pictilisib (GDC-0941) [135]. CUDC-907 through its integrated HDAC inhibitory activity induces lasting inhibition of PI3K-Akt-mTOR pathway and compensatory signaling molecules such as Raf, MEK, MAPK, and STAT-3, and upstream receptor tyrosine kinases as assessed by western blotting. Dose-dependent sustained downregulated phosphorylation of Akt and its downstream targets, 4EBP-1, and p70S by CUDC-907 in H460 cells not only confirms the PI3K pathway inhibitory activity of CUDC-907 but also exhibits the potential to evade resistance stemming from downstream activation of Akt. CUDC-907 also induced the accumulation of acetylated histone H3, tubulin, and p53, the effects specific to HDAC inhibition. CUDC-907 displayed greater growth inhibition and proapoptotic activity than single-target HDAC and PI3K inhibitors in both cultured and implanted cancer cells of xenograft tissue. Taken together, CUDC-907 evades drug resistance, stimulates apoptosis, and induces cell cycle arrest.

Additionally, oral administration of CUDC-907 at a dose of 100 mg/Kg induced tumor regression or stability in Daudi non-Hodgkin lymphoma, SU-DHL-4 diffuse large B-cell lymphoma (DLBCL), and KRAS-mutant A549 NSCLC xenografts with decent tolerance. A phase I study to evaluate the safety, tolerability, and preliminary effectiveness of CUDC-907 in patients with refractive or relapsed lymphoma or multiple myeloma was performed. Following the expected objective, several phase II clinical studies of CUDC-907 in patients with DLBCL and patients with advanced thyroid cancer are currently in process [134].

CUDC-907 also exhibits promising therapeutic potential in MYC-dependent cancers such as B-cell lymphoma [138,139]. The anticancer effect of CUDC-907 was tested in different lymphoma cell lines, which revealed effective inhibition of growth and proliferation and stimulation of cell death. Furthermore, CUDC-907 inhibited phosphorylation of PI3K downstream targets including Akt, PRAS40, S6, 4EBP-1, increased histone H3 acetylation, and decreased MYC expression. CUDC-907 also exhibited in vivo efficacy in a xenograft mouse model with no significant toxicity. These observations indicate a mechanistic rationale for assessing the therapeutic potential of CUDC-907 in MYC and PI3K-dependent lymphomas.

In AML patients PI3K/Akt pathway is constitutively active and preclinical studies demonstrate HDACIs enhance the effectiveness of PI3KIs. This observation prompted to explore the therapeutic potential of CUDC-907 in the treatment of AML [141]. CUDC-907 induces apoptosis in AML cell lines and primary AML samples and shows in vivo efficacy in an AML cell-line-derived xenograft mouse model. CUDC-907-induced apoptosis was partially dependent on Mcl-1, Bim, and c-Myc, but downregulation of CHK1, Wee1, and RRM1 and induction of DNA damage contributed to CUDC-907-induced apoptosis of AML cells [141].

**Figure 5:**
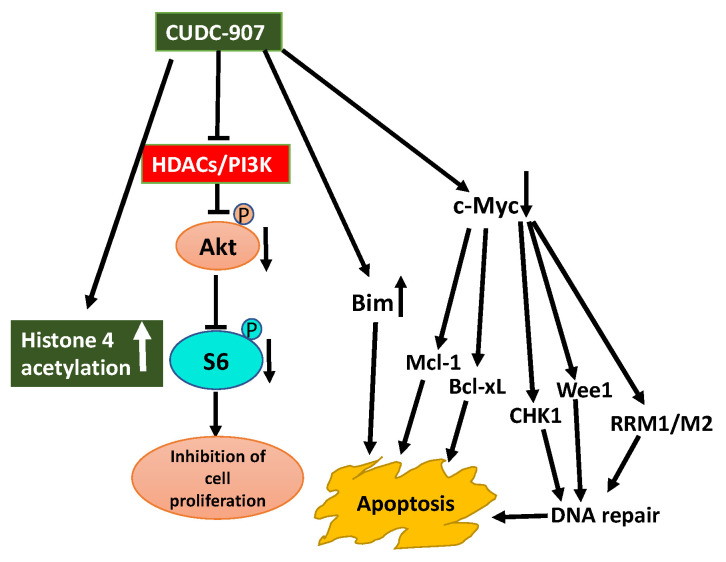
Schematic display of antitumor activity of CUDC-907 hybrid drug against prostate cancer cells. Synergistic anti-cancer activity of CUDC-907 is illustrated by its inhibition of HDACs activity and PI3K/Akt pathway [144]. Increase in histone 4 acetylation and reduced activation of PI3K/Akt confirms its dual action on HDAC and PI3K. Outcome of these effects resulted in inhibition of cell proliferation, increase in DNA damage due to downregulation of DNA damage repair response proteins, and apoptosis because of downregulation of c-myc and antiapoptotic Mcl-1 and Bcl-xL proteins, and upregulation of pro-apoptosis protein Bim (blunt end lines indicate inhibition, upward arrows indicate upregulation and downward arrows indicate downregulation of proteins).

Aberrant expression of HDACs and activation of the PI3K/Akt pathway are characteristic features of prostate cancer. Inhibition of these aberrant activities may offer therapeutic options for prostate cancer. Use of CUDC-907 displayed promising antitumor activity against prostate cancer cell lines in vitro and castration-resistant LuCaP 35CR patient-derived xenograft (PDX) mouse model in vivo (Figure 5) [144]. The antitumor activity of CUDC-907 was first determined by its effect on Akt/mTOR and ERK, pathways downstream of HDACs, and PI3K. Treatment of prostate cancer cells with CUDC-907 increased histone H4 acetylation confirming HDAC inhibitory activity of CUDC-907. Decreased phosphorylation of Akt and S6 in CUDC-907 treated cells substantiating PI3K inhibitory activity of CUDC-907. However, CUDC-907 treatment had no inhibitory effect on the activation of ERK1/2 considering their role in cell proliferation. This response may represent resistance to CUDC-907. Furthermore, an assessment of the molecular mechanism linked to the antitumor activity of CUDC-907 demonstrates that it involves induction of apoptosis and DNA damage. CUDC-907 treatment-induced decreased antiapoptotic Mcl-1 and Bcl-xL levels, increased proapoptotic Bim and decreased *c-myc* contribute to the onset of apoptosis. CUDC-907 also induced DNA damage and apoptosis by downregulating DNA damage response proteins like Wee1, CHK1, RRM1, and RRM2 (Figure 5). Moreover, the treatment of LuCaP 35CR patient-derived xenograft with CUDC-907 resulted in significant inhibition of tumor growth encouraging further clinical development of CUDC-907 for prostate cancer treatment [144].

**Figure 6:**
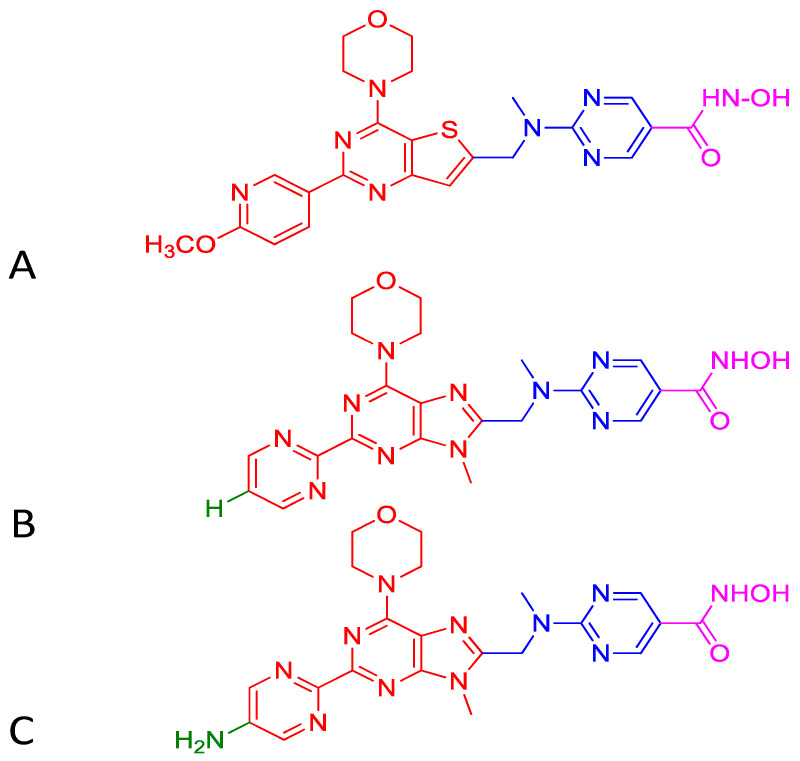
Structure of CUDC-907 (**A**) and the two active analogs (**B**) and (**C**). Morpholinopyrimidine moiety of PI3K pharmacophore (shown in red) in Figure (**A**) is integrated to HDAC inhibitory hydroxamic acid functionality (shown in magenta) through a linker (shown in blue). In Figure (**B**,**C**), morpholinopyrimidine moiety shown in Figure (**A**) is replaced with morpholinopurine (shown in red). The analog having a pyrimidine with hydrogen (green) at the C-2 position of the morpholinopurine moiety is shown in Figure **B**. The analog having a pyrimidine with an amino group (green) at the C-2 position of the morpholinopurine moiety is shown in Figure (**C**).

Upregulation of class I HDACs is a common feature in hepatocellular carcinoma (HCC), which sponsors spreading, aggressiveness, and increased mortality. Motivated by the efficacious activity of CUDC-907, efforts were made to design novel HDAC/PI3K dual hybrid drugs via bio-isosterism, by replacing morpholinothienopyrimidine moiety (Figure 6A) in CUDC-907 with a morpholinopurine (Figure 6B,C) [137]. All compounds generated displayed strong anti-HDAC1 and anti-PI3Kα activities. CUDC-907 exhibited IC_50_ values of 1.7 and 19 nM for HDAC1 and PI3Kα, respectively (Figure 6A). Among the several analogs obtained, the compounds having a pyrimidine (Figure 6B) or aminopyrimidine (Figure 6C) at the C-2 position of the morpholinopurine moiety, demonstrated expected dual PI3Kα and HDAC1 inhibitory activities. The IC_50_ values for the analog in Figure 6B against HDAC1 and PI3Kα were 1.14 and 28.06 nM, respectively. Analog in Figure 6C displayed lower and balanced IC_50_ values for HDAC1 and PI3Kα, 1.04, and 1.33 nM, respectively.

Using the polypharmacology-based paradigm, two other dual HDAC-PI3K inhibitors were recently reported [143]. These inhibitors were designed by introducing hydroxamic acid moiety as the zinc binding functional group to a quinazoline-based PI3K pharmacophore through an appropriate linker. Nanomolar concentrations of both these lead inhibitors simultaneously inhibit PI3K and HDAC activity. These inhibitors inhibited proliferation, arrested the cell cycle, and induced apoptosis of HCT116 cancer cells. Furthermore, they also altered p-Akt expression and acetylated histone H3 in HCT116. Pharmacokinetic studies in HCT116 and HGC-27 xenograft models further demonstrate that they have significant anticancer efficacies. Taken together, this study further supports the dual HDAC-PI3K hybrid drugs that have potential anticancer activity.

## 6. Challenges and Concerns

Treating multifactorial diseases such as cancer with genetic and epigenetic aberrations is challenging due to alterations in multiple biological pathways. The ability of HDACIs to cause cell cycle arrest and cell death across a broad spectrum of different types of hematologic and solid tumor types has resulted in vigorous investigations on these drugs as promising anticancer agents. A systematic understanding of the effect of these inhibitors promotes an appropriate therapeutic approach for anticancer therapy. Currently, several HDACIs are in clinical trials both in monotherapy and in combination therapy with other drugs. As single agents, they have shown promise in the treatment of hematologic malignancies, but their effect in solid tumors is not met with good success potentially due to acquired drug resistance, insufficient efficacy, toxicity, and off-target effects. To overcome these issues, HDACIs can be used in combination with other drugs or as single hybrid drugs fused with other therapeutic agents via linked pharmacophores [15,16,134]. Since cancer is the outcome of complex multifactorial genetic and epigenetic alterations, drug combinations or single multitarget hybrid drug molecules have a better opportunity to modify or reverse the altered disease-related molecular pathways. To qualify for the drug combination or multitarget hybrid molecule-based therapy, the single drugs still needs to be evaluated for their effective doses, toxicity profile, and proof of inhibitor efficacy through in-vitro assays and preclinical testing. This is because synergistic or additive biological effects of drug combinations can only be assessed by the final disease response.

Drug combination approach requiring administration of two or more drugs separately, each with their own single target (multi medication therapy, MMT) or multi medications co-formulated as a single tablet (multi-compound medication, MCM) is expected to have synergistic or additive effects. The MMT approach can provide therapeutic efficacy at a lower dose of each drug because of the synergistic effects of the drug combination. As a result, the MMT approach can reduce the chances of drug resistance and adverse reactions compared to single-drug treatment [15,16]. While the MMT approach provides flexibility to choose different dosages and therapeutic schedules, it also causes problems with patient compliance. The MCM approach can offset the negative impact on patient compliance by providing multi-medications in the form of a co-formulated single tablet [16]. However, both MMT and MCM approaches present several challenges. The use of drugs with very different chemical structures in the same therapeutic drug combination complicates pharmacodynamic and pharmacokinetic requirements. Other problems include drug–drug interactions and potential solubility issues of the active substances that can modify the bloodstream uptake. Similarly, the simultaneous presence of drugs in the target tissues is not guaranteed to promote a synergistic response. Additional complications include each tumor and the therapeutic response of each patient is different.

Unlike drug combination approaches, the use of single multitarget hybrid drugs created based on the multitarget-directed ligands (MTDLs) approach exhibits promising outcomes for solid tumors and drug-resistant cancers. The single multitarget hybrid drugs created by using the polypharmacology principle can simultaneously hit two or more targets. Importantly, single multitarget hybrid drugs have several advantages over the drug combinations. They display better pharmacokinetics and pharmacodynamics such as no solubility/bioavailability issues, greater therapeutic efficacy, lower toxicities, the simultaneous presence of the chemical entities in multiple tissues, and improved patient compliance [15,16]. Specifically, single multitarget hybrid drugs that can simultaneously hit two or more targets in which one (or more) is (are) epigenetic and the other (s) belongs to a non-epigenetic category(ies) have greater potential for cancer therapy. This concept has resulted in the design and development of multitarget epigenetic hybrid drugs. Among the several epigenetic hybrid drugs produced, CUDC-907 is a promising single dual HDAC-PI3K inhibitor molecule with an ability to simultaneously inhibit HDACs and PI3K [15,16,134,135,136,137,138,139,140,141,142,143,144]. CUDC- 907 is an orally bioavailable small -molecule dual HDAC and PI3K inhibitor targeting class I and II HDACs and PI3Kα, β, and δ isoforms [135]. Its extensive antitumor activities in hematologic and solid tumors show promise as an HDAC-PI3K inhibitor [134,135,136,137,138,139,140,141,142,143,144]. Considering the pleiotropic effects of HDAC inhibition and the multiple downstream effectors of PI3K, it is not surprising CUDC-907 has a better opportunity to reverse the altered molecular pathways linked to oncogenesis. Even with the frequent difficulty in structural optimization to effectively merge the pharmacophoric portions of HDAC and PI3K entities to generate dual HDAC-PI3K inhibitor, it was possible to generate CUDC-907. Importantly, preclinical findings indicate the use of CUDC-907 has several advantages over the drug combination treatment with single-target HDAC and PI3K inhibitors (MMT and MCM) [15,16,135]. CUDC-907 exhibits better pharmacokinetics and pharmacodynamics such as no solubility/bioavailability issues, greater therapeutic efficacy, lower toxicities, the simultaneous presence of the chemical entities in multiple tissues, and improved patient compliance [15,16,135]. Moreover, CUDC-907 is well tolerated with manageable gastrointestinal and hematologic events compared to a combination of single HDAC and PI3K inhibitors without any infections, hepatotoxicity, or cardiotoxicity [136]. Adjusting dosing is simpler and pharmacokinetics and toxicity profiles are predictable. Based on these preclinical outcomes, CUDC907 was entered into phase I study to determine the safety, tolerability, and pharmacokinetics in patients with advanced/relapsed solid tumors (NCT02307240). Based on encouraging results from the phase I study, a biomarker-driven phase II (NCT02674750) study has been initiated in patients with *myc* mutation.

## 7. Conclusions and Perspectives

Epigenetic drug discovery is a rapidly evolving field, and the use of epigenetic drugs both in clinical and preclinical trials forecast a promising future for the treatment of multifactorial diseases such as cancer. The first dual inhibitor targeted at an epigenetic target was reported in 2007 and since then several dual inhibitors aimed at epigenetic targets have been reported. Importantly, most of these inhibitors include HDAC inhibition as one of their mechanisms of action. This is attributed to the high resilience offered by the HDAC pharmacophore. Moreover, the characteristics of HDACs and strategies for their inhibition are better understood compared to other epigenetic targets. CUDC-907 is one of the successes of the combination of HDAC inhibition with PI3K inhibition. Several other epigenetic drugs are undergoing clinical trials in combination with other therapeutic agents via linked pharmacophores that can provide synergistic effects both for solid and hematologic cancers. CUDC-907 inhibits classical HDACs and all four class I PI3K isoforms in nanomolar amounts, and is currently in phase II cancer trials. Despite the promising efficacy in hematologic cancers, there is still a poor prognosis for solid tumors due to poor cancer-targeting specificity. A recent report indicates that a nanoparticle-based pre-targeted delivery system improves the therapeutic window of small-molecule inhibitors [161]. This approach increases the number of nanoparticles retained on the target tumor cells, improves the drug delivery, and shows the antitumor activity of BEZ235 through the inhibition of PI3K/mTOR. The use of nanoparticle-based drug delivery technology may improve the delivery of CUDC-907 to target cancer tissues, and at the same time, normal tissues can be protected. The future looks bright for dual inhibitors directed at epigenetic targets and their full potential in the treatment of complex diseases is yet to be explored.

## References

[1] Vanhaesebroeck B., Guillermet-Guibert J., Graupera M., Bilanges B. (2010). The emerging mechanism of isoform-specific PI3K signaling. Nat. Rev. Mol. Cell Biol..

[2] Vanhaesebroeck B., Stephens L., Hawkins P. (2012). PI3K signaling: The path to discovery and understanding. Nat. Rev. Mol. Cell Biol..

[3] Engelman J.A., Luo J., Cantley L.C. (2006). The evolution of phosphatidylinositol 3-kinases as regulators of growth and metabolism. Nat. Rev. Genet..

[4] Lawrence M.S., Stojanov P., Mermel C.H., Robinson J.T., Garrqway L.A., Golub T.R., Meyerson M.L., Gabriel S.B., Lander E.S., Getz G. (2014). Discovery and saturation analysis of cancer genes across 21 tumour types. Nature.

[5] Gyori D., Chessa T., Hawkins P.T., Stephens L.R. (2017). Class (I) phosphoinositide 3-kinases in the tumor microenvironment. Cancers.

[6] Thorpe L.M., Yuzugullu H., Zhao J.J. (2015). PI3K in cancer: Divergent roles of isoforms, modes of activation and therapeutic targeting. Nat. Rev. Cancer.

[7] Janku F., Yap T., Meric-Bernstam F. (2018). Targeting the PI3K pathway in cancer: Are we making head way?. Nat. Rev. Clin. Oncol..

[8] McCubery J.A., Steelman L.S., Chappell W.H., Abrams S.L. (2012). Franklin, Ras/Raf/MEK/ERK and PI3K/PTEN/Akt/mTOR cascade inhibitors: How mutations can result in therapy resistance and how to overcome resistance. Oncotarget.

[9] Wee S., Jagani Z., Xiang K.X., Loo A. (2009). PI3k pathway activation mediates resistance to MEK inhibitors in KRAS mutant cancers. Cancer Res..

[10] Yang X.J., Seto E. (2007). HATs and HDACs: From structure, function and regulation to novel strategies for therapy and prevention. Oncogene.

[11] Hagelkruys A., Sawicka A., Rennmayr M., Seiser C. (2011). The biology of HDAC in cancer: The nuclear and epigenetic components. Handb. Exp. Pharmacol..

[12] Delcuve G.P., Khan D.H., Davie J.R. (2012). Roles of histone deacetylases in epigenetic regulation: Emerging paradigms from studies with inhibitors. Clin. Epigenet..

[13] Zwergel C., Stazi G., Valente S., Mai A. (2016). Histone Deacetylase Inhibitors: Updated Studies in Various Epigenetic-Related Diseases. J. Clin. Epigenet..

[14] Benedetti R., Conte M., Iside C., Atlcci L. (2015). Epigenetic-based therapy: From single- to multi-target approaches. Int. J. Biochem. Cell Biol..

[15] deLera A.R., Ganesan A. (2016). Epigenetic polypharmacology: From combination therapy to multitarget drugs. Clin. Epigenet..

[16] Tomaselli D., Lucidi A., Rotili D., Mai A. (2020). Epigenetic polypharmacology: A new frontier for epi-drug discovery. Med. Res. Rev..

[17] Okkenhaug K. (2013). Signaling by the phosphoinositide 3-kinase family in immune cells. Annu. Rev. Immunol..

[18] Cantley L.C. (2002). The phosphoinositide 3-kinase pathway. Science.

[19] Rai S.N., Dilnashin H., Birla H., Singh S.S., Zahra W., Rathore A.S., Singh B.K., Singh S.P. (2019). The role of PI3K/Akt and ERK in neurodegenerative disorders. Neurotox Res..

[20] Gross C., Bassell G.J. (2014). Neuron-specific regulation of class I PI3K catalytic subunits and their dysfunction in brain disorders. Front. Mol. Neurosci..

[21] Oudit G.Y., Sun H., Kerfant B.-G., Crackower M.A., Penninger J.M., Backk P.H. (2004). The role of phoisphoinositide-3 kinase and PTEN in cardiovascular physiology and disease. J. Mol. Cell. Cardiol..

[22] Eisenreich A., Rauch U. (2011). PI3K inhibitors in cardiovascular disease. Cardiovasc. Ther..

[23] Durrant T.N., Hers I. (2020). PI3K inhibitors in thrombosis and cardiovascular disease. Clin. Trans. Med..

[24] Maffei A., Lembo G., Carnevale D. (2018). PI3Kinases in diabetes mellitus and its related complications. Int. J. Mol. Sci..

[25] Kurek K.C., Luks V.L., Ayturk U.M., Alomari A.I., Fishman S.J., Spencer S.A., Mulliken J.B., Bowen M.E., Yamamoto G.L., Kozakewich H.P.W. (2012). Somatic mosaic activating mutations in PIK3CA cause CLOVES syndrome. Am. J. Hum. Genet..

[26] Parsons R. (2004). Human cancer, PTEN, and the PI-3 kinase pathway. Semin. Cell Dev. Biol..

[27] Song M.S., Salmena L., Pandolfi P.P. (2012). The functions and regulation of the PTEN tumour suppressor. Nat. Rev. Mol. Cell Biol..

[28] Aziz S.A., Davies M., Pick E., Zito C., Jilaveanu L., Camp R.L., Rimm D.L., Kluger Y., Kluger H.M. (2009). Phosphatidylinositol 3-kinase as a therapeutic target in melanoma. Clin. Cancer Res..

[29] Zhou B.P., Hu M.C., Miller S.A., Yu Z., Xia W., Lin S.Y., Hung M.C. (2000). Her-2/neu blocks tumor necrosis factor-induced apoptosis via the Akt/NF-kappaB pathway. J. Biol. Chem..

[30] Hill K., Kalifa S., Das J.R., Bhatti T., Gay M., Williams D., Taliferro-Smith L., De Marzo A.M. (2010). The role of PI 3-kinase p100beta in Akt signaling, cell survival, and proliferation in human prostate cancer cells. Prostate.

[31] Dbouk H., Khalil B.D., Wu H., Shymanets A., Nurnberg B., Backer J.M. (2013). Characterization of tumor-associated activating mutation of the p110β PI 3-K. PLoS ONE.

[32] Urick M.E., Rudd M.L., Godwin A.K., Sgroi D., Merino M., Bell D.W. (2011). PIK3R1 (p85alpha) is somatically mutated at high frequency in primary endometrial cancer. Cancer Res..

[33] Wu H., Shekar S.C., Flinn R.J., El-Sibai M., Jaiswal B.S., Sen K.L., Janakiraman V., Seshagiri S., Gerfer G.J., Girvin M.E. (2009). Regulation of class IA PI3-kinases: C2 domain- iSH2 domain contacts inhibit p85/p110alpha and are disrupted in oncogenic p85 mutants. Proc. Natl. Acad. Sci. USA.

[34] Cortes I., Sanchez-Ruiz J., Zuluaga S., Calvanese V., Marques M. (2012). p85beta phosphoinositide 3-kinase subunit regulates tumor progression. Proc. Natl. Acad. Sci. USA.

[35] Engelman J.A. (2009). Targeting PI3K signaling in cancer: Opportunities, challenges and limitations. Nat. Rev. Cancer.

[36] Spangle J.M., Roberts T.M., Zhao J.J. (2017). The emerging role of PI3K/Akt-mediated regulation in cancer. Biochem. Biophys. Acta.

[37] Furman R.R., Sharman J.P., Coutre S.E., Cheson B.D., Pagel J.M., Hillmen P., Barrientos J.C., Zelenetz A.D., Kipps T.J., Flinn I. (2014). Idelalisib and rituximab in relapsed chronic lymphocytic leukemia. N. Engl. J. Med..

[38] Ramesh L.E., Chen C.S., Cantley L.C. (1995). Phosphatidylinositol (3,4,5)P3 interacts with SH2 domains and modulates PI 3-kinase association with tyrosine-phosphorylated proteins. Cell.

[39] Yu J., Wjasow C., Backer J.M. (1998). Regulation of p85/p110alpha phosphatidylinositol 3-kinase. Distinct roles for the n-terminal and c-terminal SH2 domains. J. Biol. Chem..

[40] Manning B.D., Toker A. (2017). Akt/PKB signaling: Navigating the network. Cell.

[41] Milella M., Falcone I., Conciatori F., Incani U.C., Curatolo A.D., Inzerilli N., Nuzzo C.M.A., Vaccaro N., Vari S., Cognetti F. (2015). PTEN: Multiple functions in human malignant tumors. Front. Oncol..

[42] Ong P.S., Wang L.Z., Dai X., Tseng S.H., Loo S.J., Sethi G. (2016). Judicious toggling of mTOR activity to combat insulin resistance and cancer: Current evidence and perspectives. Front. Pharmacol..

[43] Shahbazian M.D., Grunstein M. (2007). Functions of site-specific histone acetylation and deacetylation. Annu. Rev. Biochem..

[44] Lee K.K., Workman J.L. (2007). Histone acetyltransferase complexes: One size doesn’t fit all. Nat. Rev. Mol. Cell Biol..

[45] de Ruijter A.J., van Gennip A.H., Caron H.N., Kemp S., van Kuilenburg A.B. (2003). Histone deacetylases (HDACs): Characterization of the classical HDAC family. Biochem. J..

[46] Clayton A.L., Hazzalin C.A., Mahadevan L.C. (2006). Enhanced histone acetylation and transcription: A dynamic perspective. Mol. Cell.

[47] Choudhary C., Kumar C., Gnad F., Nielsen M.L., Rehman M., Walther T.C., Olsen J.V., Mann M. (2009). Lysine acetylation targets protein complexes and co-regulates major cellular functions. Science.

[48] Ranganna K., Yatsu F.M., Mathew O.P., Parthasarathy S. (2012). Emerging epigenetic therapy for vascular proliferative diseases. Atherogenesis.

[49] Ranganna K., Mathew O.P., Milton S.G., Li C. (2014). Regulation of cellular processes by epigenetic mechanisms of butyrate. Butyrate: Food Sources, Functions and Health Benefits. Biochemistry Research Trends Series.

[50] Latham J.A., Dent S.Y. (2007). Cross-regulation of histone modifications. Nat. Struct. Mol. Biol..

[51] Yang X.J., Seto E. (2008). Lysine acetylation: Codified crosstalk with other posttranslational modifications. Mol. Cell.

[52] Wang Q., Zhang Y., Yang C., Xiong H., Lin Y., Yao J., Li H., Xie L., Zhao W., Yao Y. (2010). Acetylation of metabolic enzymes coordinates carbon source utilization and metabolic flux. Science.

[53] Patel J., Pathak R.R., Mujtaba S. (2011). The biology of lysine acetylation integrates transcriptional programming and metabolism. Nutr. Metab..

[54] Cheung P., Allis C.D., Sassone-Corsi P. (2000). Signaling to chromatin through histone modifications. Cell.

[55] Strahl B.D., Allis C.D. (2000). The language of covalent histone modifications. Nature.

[56] Kouzarides T. (2007). Chromatin modifications and their function. Cell.

[57] Mathew O.P., Ranganna K., Yatsu F.M. (2010). Butyrate, a histone deacetylase inhibitor, stimulates interplay between different posttranslational modifications of histone H3 and differently alters G1-specific cell cycle proteins in vascular smooth muscle cells. Biomed. Pharmacother..

[58] Gil J., Ramirez-Torres A., Encarnacion-Guevara S. (2017). Lysine acetylation and cancer: A proteomics perspective. J. Proteom..

[59] Grimes M., Benjamin H., Foltz L., Levy T., Rikova K., Gaiser J., Cook W., Smirnova E., Wheeler T., Clark N.R. (2018). Integration of protein phosphorylation, acetylation and methylation data sets to outline lung cancer signaling network. Sci. Signal..

[60] Alonso-Bastda R., Encarnacion-Guevara S. (2019). Proteomic insights into lysine acetylation and the implications for medical research. Exptert Rev. Proteom..

[61] Okumura K., Mendoza M., Bachoo R.M., DePinho R.A., Cavenee W.K., Fumari F.B. (2006). PCAF modulates PTEN activity. J. Biol. Chem..

[62] Buuh Z.Y., Lyu Z., Wang R.E. (2017). Interrogating the roles of post-translational modifications of non-histone proteins. J. Med. Chem..

[63] Filippakopoulos P., Knapp S. (2014). Targeting bromodomains: Epigenetic readers of lysine acetylation. Nat. Rev. Drug Discov..

[64] Perez-Salvia M., Esteller M. (2017). Bromodomain inhibitors and cancer therapy: From structures to applications. Epigenetics.

[65] Di Cerbo V., Schneider R. (2013). Cancers with wrong HATs: The impact of acetylation. Briefings Funct. Genom..

[66] Sambataro F., Pennuto M. (2017). Post-translational modifications and protein quality control in motor neuron and polyglutamine diseases. Front. Mol. Neurosci..

[67] Liu J., Qian C., Cao X. (2016). Post-translational modification control of innate immunity. Immunity.

[68] Riggs M.G., Whittaker R.G., Neumann J.R., Ingram V.M.N. (1977). Butyrate causes histone modification in Hela and Friend erythroleukemia cells. Nature.

[69] Xu W.S., Parmigiani R.B., Marks P.A. (2007). Histone deacetylase inhibitors: Molecular mechanism of action. Oncogene.

[70] Garmpi A., Garmpis N., Damaskos C., Valsami S., Spartalis E., Lavaris A., Patelis N., Margonis G.-A., Apostolou K.G., Sparatalis M. (2018). Histone deacetylase inhibitors as a new anticancer option: How far can we go with expectations?. J. BUON.

[71] Chen C.-S., Weng S.-C., Tseng P.-H., Chen C.-S. (2005). Histone acetylation-independent effect of histone deacetylase inhibitors on Akt through the reshuffling of protein phosphatase 1 complexes. J. Biol. Chem..

[72] Wang J.C., Kafeel M.I., Avezbakiyev B., Chen C., Sun Y., Rathnasabapathy C., Kalavar M., He Z., Barton J., Lichter S. (2011). Histone deacetylase in chronic lymphocytic leukemia. Oncology.

[73] Zhang H., Shand Y.P., Chen H.Y., Li J. (2017). Histone deacetylases function as novel potential therapeutic targets for cancer. Hepatol. Res..

[74] Zhang Q., Wang S., Chen J., Yu Z. (2019). Histone deacetylases (HDACs) guided novel therapies for T-cell lymphomas. Int. J. Med. Sci..

[75] Quagliano A., Gopalakrihnapillai A., Barwe S.P. (2020). Understanding the mechanisms by which epigenetic modifiers avert therapy resistance in cancer. Front. Oncol..

[76] Baylin S.B., Jones P.A. (2011). A decade of exploring the cancer epigenome biological and translational implication. Nat. Rev. Cancer.

[77] Fraga M.F., Ballestar E., Villar-Garea A., Boix-Chornet M., Espada J., Scotta G., Bonaldi T., Haydon C., Ropero S., Petrie K. (2005). Loss of acetylation at lys16 and trimethylation of lys20 of histone H4 is a common hallmark of human cancer. Nat. Genet..

[78] Zeller C., Brown R. (2010). Therapeutic modulation of epigenetic drivers of drug resistance in ovarian cancer. Ther. Adv. Med. Oncol..

[79] Peart M.J., Smyth G.K., Van Laaar R.K., Bowtell D.D., Richon V.M., Marks P.A., Holloway A.J., Johnstone R.W. (2005). Identification and functional significance of genes regulated by structurally different histone deacetylase inhibitors. Proc. Natl. Acad. Sci. USA.

[80] Smith K.T., Workman J.L. (2009). Histone deacetylase inhibitors: Anticancer compounds. Int. J. Biochem. Cell. Biol..

[81] Verza F.A., Das U., Fachin A.L., Dimmock J.R., Marins M. (2020). roles of histone deacetylases and inhibitors in anticancer therapy. Cancers.

[82] Bolden J.E., Peart M.J., Johnstone R.W. (2006). Anticancer activities of histone deacetylase inhibitors. Nat. Rev. Drug Discov..

[83] Stazi G., Fioravanti R., Mai A., Mattevi A., Valente S. (2019). Histone deacetylases as an epigenetic pillar for the development of hybrid inhibitors in cancer. Curr. Opin. Chem. Biol..

[84] Blagosklonny M.V., Robey R., Sackett D.L., Du L., Traganos F., Darzynkiewicz Z., Fojo T., Bates S.E. (2002). Histone deacetylase inhibitors all induce p21 but differently cause tubulin acetylation, mitotic arrest, and cytotoxicity. Mol. Cancer Ther..

[85] Susanto J.M., Colvin E.K., Pinese M., Chang D.K., Pajic M., Mawson A., Caldon C.E., Musgrove E.A., Henshall S.M., Sutherland R.L. (2015). The epigenetic agents suberoylanilide hydroxamic acid and 5AZA2′ deoxycytidine decrease cell proliferation, induce cell death and delay the growth of MiaPaCa2 pancreatic cancer cells in vivo. Int. J. Oncol..

[86] Bali P., Pranpat M., Swaby R., Fiskus W., Yamaguchi H., Balasis M., Rocha K., Wang H.-G., Richon V., Bhalla K. (2005). Activity od suberoylanilide hydroxamic acid against human breast cancer cells with amplification of her-2. Clin. Cancer Res..

[87] Yeh C.C., Deng Y.T., Sha D.Y., Hsiao M., Kuo M.Y. (2009). Suberoylanilide hydroxamic acid sensitizes human oral cancer cells to TRAIL-induced apoptosis through increase DR5 expression. Mol. Cancer Ther..

[88] Mrakovcic M., Frohlich L.F. (2020). Molecular determinants of cancer therapy resistance to HDAC inhibitor-induced autophagy. Cancers.

[89] Deng B., Luo Q., Halim A., Liu Q., Zhang B., Song G. (2020). The antiangiogenesis role of histone deacetylases inhibitors: Their potential application to tumor therapy and tissue repair. DNA Cell Biol..

[90] Zhang J., Zhong Q. (2014). Histone deacetylase inhibitors and cell death. Cell. Mol. Life Sci..

[91] Schiattarella G.G., Sannino A., Toscano E., Cattanco F., Trimarco B., Esposito G., Perrino C. (2016). Cardiovascular effects of histone deacetylase inhibitors epigenetic therapies: Systemic review of 62 studies and new hypothesis for future research. Int. J. Cardiol..

[92] Meng J., Li Y., Camarillo C., Yao Y., Zhang Y., Xu C., Jiang L. (2014). The anti-tumor histone deacetylase inhibitor SAHA and the natural flavonoid curcumin exhibit synergistic neuroprotection against amyloid-beta toxicity. PLoS ONE.

[93] Jonsson K.L., Tolstrup M., Vad-Nielsen J., Kjaer K., Laustsen A., Andersen M.N., Rasmussen T.A., Sogaard O.S., Ostergaard L., Denton P.W. (2015). Histone deacetylase inhibitor romidepsin inhibits de novo HIV-1 infections. Antmicrob. Agents Chemther..

[94] Wiech N.I., Fisher J.F., Helquist P., Wiest O. (2009). Inhibition of histone deacetylases: A pharmacological approach to the treatment of non-cancer disorders. Curr. Top. Med. Chem..

[95] Merarchi M., Sethi G., Shanmugam M.K., Fan L., Arfuso F., Ahn K.S. (2019). Role of natural products in modulating histone deacetylase in cancer. Molecules.

[96] Marson C.M. (2009). Histone deacetylase inhibitors: Design, structure-activity relationships and therapeutic implications for cancer. Anti-Cancer Agents Med. Chem..

[97] West A.C., Johnstone R.W. (2014). New and emerging HDAC inhibitors for cancer treatment. J. Clin. Invest..

[98] Drummond D.C., Noble C.O., Kirpotin D.B., Guo Z., Scott G.K., Benz C.C. (2005). Clinical development of histone deacetylase inhibitors as anticancer agents. Annu. Rev. Pharmacol. Toxicol..

[99] Stenzel K., Hamacher A., Hansen F.K., Gertzen C.G.W., Senger J., Marquart V., Marek L., Marek M., Romier C., Remke M. (2017). Alkoxyurea-based histone deacetylase inhibitors increase cisplatin potency in chemoresistant cancer cell lines. J. Med. Chem..

[100] Coiffier B., Pro B., Prince H.M., Foss F., Sokol L., Greenwood M., Caballero D., Borchmann P., Morschhauser F., Wilhelm M. (2012). Results from a pivotal, open-label, phase II study of romidepsin in relapsed or refractory peripheral T-cell lymphoma after prior systemic therapy. J. Clin. Oncol..

[101] Qiu T., Zhou L., Zhu W., Wang T., Wang J., Shu Y., Liu P. (2013). Effects of treatment with histone deacetylase inhibitors in solid tumors: A review based on 30 clinical trials. Future Oncol..

[102] Rosik L., Niegish G., Fischer U., Jung M., Schulz W.A., Hoffmann M.J. (2014). Limited efficacy of specific HDAC6 inhibition in urothelial cancer cells. Cancer Biol. Ther..

[103] Tiash S., Chowdhary E. (2015). Growth factor receptors: Promising drug targets in cancer. J. Cancer Metastasis Treat..

[104] Yang J., Nie J., Ma X., Wei Y., Peng Y., Wei X. (2019). Targeting PI3K in cancer: Mechanisms and advances in clinical trials. Mol. Cancer.

[105] Fuino L., Bali P., Whttmann S., Donapaty S., Guo F., Yamaguchi H., Wang H.-G., Atadja P., Bhalla K. (2003). Histone deacetylase inhibitor LAQ824 down-regulates Her-2 and sensitizes human breast cancer cells to trastuzumab, taxotere, gemcitabine, and epothilone B. Mol. Cancer Ther..

[106] Lee J., Bartholomeusz C., Mansour O., Hhmphries J., Hortobagyi G.N., Ordentlich P., Ueno N.T. (2014). A class I histone deacetylase inhibitor, entinostat, enhances lapatinib efficacy in HER2-overexpressing breast cancer cells through FOXO3-mediated Bim 1 expression. Breast Cancer Res. Treat..

[107] von Karstedt S., Montinaro A., Walczak H. (2017). Exploring the TRAILs less traveled: TRAIL in cancer biology and therapy. Nat. Rev. Cancer.

[108] Butler L.M., Liapis V., Bouralexis S., Welldon R., Hay S., Thai L.M., Labrinidis A., Tilley W.D., Findlay D.M., Evdokiou A. (2006). The histone deacetylase inhibitor, suberoylanilide hydroxamic acid, overcomes resistance of human breast cancer cells to Apo2L/TRAIL. Int. J. Cancer.

[109] Frew A.J., Lindaemann R.K., Martin B.P., Clarke C.J.P., Sharkey J., Anthony D.A., Banks K.-M., Haynes N.M., Gangatirkar P., Stanley K. (2008). Combination therapy of established cancer using a histone deacetylase inhibitor and a TRAIL receptor agonist. Proc. Natl. Acad. Sci. USA.

[110] Suraweera A., O’Byrne K.J., Richard D.J. (2018). Combination therapy with histone deacetylase inhibitors (HDACi) for the treatment of cancer: Achieving the full therapeutic potential of HDACi. Front. Oncol..

[111] Fantin V.R., Richon V.M. (2007). Mechanisms of resistance to histone deacetylase inhibitors and their therapeutic implications. Clin. Cancer Res..

[112] Richon V.M., Sandhoff T.W., Rifkind R.A., Marks P.A. (2000). Histone deacetylase inhibitor selectively induces p21Waf1 expression and gene-associated histone deacetylation. Proc. Natl. Acad. Sci. USA.

[113] Sandor V., Senderowicz A., Mertins S., Sackett D., Sausville E., Blagosklonny M.V., Bates S.E. (2000). P21-deficient G(1) arrest with downregulation of cyclin D1 and upregulation of cyclin E by the histone deacetylase inhibitor FR901228. Br. J. Cancer.

[114] Gartel A.L. (2005). The conflicting roles of the cdk inhibitor p21(WAF1) in apoptosis. Leuk. Res..

[115] Vrana J.A., Decker R.H., Johnson C.R. (1999). Induction of apoptosis in U937 human leukemia cells by suberoylanilide hydroxamic acid (SAHA) proceeds through pathways that are regulated by Bcl-2/Bcl-XL, c-jun and p21Cip1, but independent of p53. Oncogene.

[116] Louis M., Rosato R.R., Brault L., Osbid S., Battaglia E., Yang X.-H., Grant S., Bagrel D. (2004). The histone deacetylase inhibitor sodium butyrate induces breast cancer cell apoptosis through diverse cytotoxic actions including glutathione depletion and oxidative stress. Int. J. Oncol..

[117] Ellis L., Bots M., Lindemann R.K., Bolden J.E., Newbold A., Cluse L.A., Scott C.L., Strasser A., Atadja P., Lowe S.W. (2009). The histone deacetylase inhibitors LAQ824 and LBH589 do not require death receptor signaling or a functional apoptosome to mediate tumor cell death or therapeutic efficacy. Blood.

[118] Newbold A., Lindemann R.K., Cluse L.A., Whitecross K.F., Dear A.E., Johnstone R.W. (2008). Characterization of the novel apoptotic and therapeutic activities of the histone deacetylase inhibitor romidepsin. Mol. Cancer Ther..

[119] Shao W., Growney J.D., Feng Y., O’Connor G., Pu M., Zhu W., Yao Y.-M., Kwon P., Fawell S., Atadja P. (2010). Activity of deacetylase inhibitor panobinostat (LBH589) in cutaneous T-cell lymphoma models: Defining molecular mechanisms of resistance. Int. J. Cancer.

[120] Inoue S., Walewska R., Dyer M.J., Cohen G.M. (2008). Downregulation of Mcl-1 potentiates HDACI-mediated apoptosis in leukemic cells. Leukemia.

[121] Yu C., Friday B.B., Lai J.P., McCollum A., Atadja P. (2007). Abrogation of MAPK and Akt signaling by AEE788 synergistically potentiates histone deacetylase inhibitor induced apoptosis through reactive oxygen species generation. Clin. Cancer Res..

[122] Fantin V.R., Loboda A., Paweletz C.P., Hendrickson R.C., Pierce J.W., Roth J.A., Li L., Gooden F., Korenchuk S., Hou X.S. (2008). Constitutive activation of signal transducer and activators of transcription predicts vorinostat resistance in cutaneous T-cell lymphoma. Cancer Res..

[123] Staudt L.M. (2010). oncogenic activation of NF-kappaB. Cold Spring Harb. Perspect. Biol..

[124] Mayo M.W., Denlinger C.E., Broad R.M., Yeung F., Reilly E.T., Shi Y., Jones D.R. (2003). Ineffectiveness of histone deacetylase inhibitors to induce apoptosis involves the transcriptional activation of NF-kB through the Akt activity. J. Biol. Chem..

[125] Rundall B.K., Delinger C.E., Jones D.R. (2004). Combined histone deacetylase and NF-kB inhibition sensitizes non-small cell lung cancer to cell death. Surgery.

[126] Dai Y., Rahmani M., Dent P., Grant S. (2005). Blockade of histone deacetylase inhibitor-induced RelA/p65 acetylation and NF-kB activation potentiates apoptosis in leukemia cells through a process mediated by oxidative damage, XIAP down-regulation, and c-Jun N-terminal kinase 1 activation. Mol. Cell. Biol..

[127] Morin R.D., Mendez-Lago M., Mungall A.J., Goya R., Mungall K.L., Corbett R.D., Johnson N.A., Severson T.M., Chiu R., Field M. (2011). Frequent mutation of histone- modifying genes in non-Hodgkin lymphoma. Nature.

[128] Chen J., Ghazawi F.M., Bakker W., Li Q. (2006). Valproic acid and butyrate induce apoptosis in human cancer cells through inhibition of gene expression of Akt/protein kinase B. Mol. Cancer.

[129] Luu T.H., Morgan R.J., Leong L., Lim D., McNamara M., Portnow J., Frankel P., Smith D.D., Doroshow J.H., Gandara D.R. (2008). A phase II trial of Vorinostat (suberoylanilide hydroxamic acid) in metastatic breast cancer: A California cancer consortium study. Clin. Cancer Res..

[130] Valdespino V., Valdespino P.M. (2015). Potential of epigenetic therapies in the management of solid tumors. Cancer Manag. Res..

[131] Ma X., Lv X., Zhang J. (2018). Exploring polypharmacology for improving outcome of kinase inhibitors (KIs): An update of recent medicinal chemistry. Eur, J. Med. Chem..

[132] Ganesan A. (2016). Multitarget drugs: An epigenetic epiphany. Chem. Med. Chem..

[133] Thum K.T., Thomas S., Moore A., Munster P.N. (2011). Rational therapeutic combinations with histone deacetylase inhibitors for the treatment of cancer. Future Oncol..

[134] Luan Y., Li J., Bernatchez J.A., Li R. (2019). Kinase and histone deacetylase hybrid inhibitors for cancer therapy. J. Med. Chem..

[135] Qian C., Lai C.-J., Bao R., Wang D.-G., Wang J., Xu G.-X., Atoyan R., Qu H., Yin L., Samson M. (2012). Cancer network disruption by a single molecule inhibitor targeting both histone deacetylase activity and phosphatidylinositol 3-kinase signaling. Clin. Cancer Res..

[136] Younes A., Berdeja J.G., Patel M.R., Flinn I., Gerecitano J.F., Neelapu S., Kelly K.R., Copeland A.R., Akins A., Clancy M.S. (2016). Safety, tolerability, and preliminary activity of CUDC-907, a first-in-class, oral, dual inhibitor of HDAC andPI3K, in patients with relapsed or refractory lymphoma or multiple myeloma: An open-label, dose escalation, phase I trial. Lancet Oncol..

[137] Chen Y., Wang X., Xiang W., He L., Tang M., Wang F., Wang T., Yang Z., Yi Y., Wang H. (2016). Development of purine-based hydroxamic acid derivatives potent histone deacetylase inhibitors with marked in vitro and in vivo antitumor activities. J. Med. Chem..

[138] Sun K., Atoyan R., Borek M.A., Dellarocca S., Samson M.E.S., Ma A.W., Xu G.-X., Patterson T., Tuck D.P., Viner J.L. (2016). Dual HDAC and PI3K inhibitor CUDC-907 downregulates MYC and suppresses growth of MYC-dependent cancers. Mol. Cancer Ther..

[139] Modello P., Derenzini E., Asgari Z., Philip J., Brea E.J., Seshan V., Henderickson R.C., de Stanchina E., Scheinberg D.A., Younes A. (2017). Dual inhibition of histone deacetylase and phosphoinositide 3-kinase enhances therapeutic activity against B-cell lymphoma. Oncotarget.

[140] Oki Y., Kelly K.R., Flinn I., Patel M.R., Gharavi R., Ma A., Parker J., Hafeez A., Tuck D., Younes A. (2017). CUDC-907 in relapsed/refractory diffuse large B-cell lymphoma, including patients with Myc-alterations: Results from an expanded phase I trial. Haematologica.

[141] Li X., Su Y., Madlambayan G., Edwards H., Polin L., Kushner J., Dzinic S.H., White K., Ma J., Knight T. (2019). Antileukemic activity and mechanism of action of the novel PI3K and histone deacetylase dual inhibitor CUDC-907 in acute myeloid leukemia. Haemtologica.

[142] Chen Y., Peubez C., Smith V., Xiong S., Kocsis-Fodor G., Kennedy B., Wagner S., Balotis C., Jayne S., Dyer M.J.S. (2019). CUDC-907 blocks multiple pro-survival signals and abrogates microenvironment protection in CLL. J. Cell. Mol. Med..

[143] Zhang K., Lai F., Lin S., Ji M., Zhang J., Zhang Y., Jin J., Fu R., Wu D., Tian H. (2019). Design, synthesis, and biological evaluation of 4-methyl quinazoline derivatives as anticancer agents simultaneously targeting phosphoinositide 3-kinases and histone deacetylases. J. Med. Chem..

[144] Hu C., Xia H., Bai S., Zhao J., Edwards H., Li X., Yang Y., Lyu J., Wang G., Zhan Y. (2020). CUDC-907, a novel dual PI3K and HDAC inhibitor, in prostate cancer: Antitumor activity and molecular mechanism of action. J. Cell. Mol. Med..

[145] Bai Z., Zhang Z., Ye Y., Wang S. (2010). Sodium butyrate induces differentiation of gastric cancer cells to intestinal cells via PTEN/phosphoinositide 3-kinase pathway. Cell Biol. Int..

[146] Ma J., Guo X., Zhang S., Liu H., Lu J., Dong Z., Liu K., Ming L. (2015). Trichostatin A, a histone deacetylase inhibitor, suppresses proliferation and promotes apoptosis of esophageal squamous cell lines. Mol. Med. Rep..

[147] Mosleh M., Safaroghli-Azar A., Bashash D. (2020). Pan-HDAC inhibitor panobinostat, as a single agent or in combination with PI3K inhibitor, induces apoptosis in APL cells: An emerging approach to overcome MSC-induced resistance. Int. J. Biochem. Cell Biol..

[148] Saijo K., Katoh T., Shimodaira H., Oda A., Takahashi O., Ishioka C. (2012). Romidepsin (FK228) and its analog directly inhibit phosphatidylinositol 3-kinase activity and potently induce apoptosis as histone deacetylase/phosphatidylinositol 3-kinase dual inhibitors. Cancer Sci..

[149] Saijo K., Imai H., Chikamatsu S., Narita K., Katoh T., Ishioka C. (2017). Antitumor activity and pharmacologic characterization of the depsipeptide analog as a novel histone deacetylase/phosphatidylinositol 3-kinase dual inhibitor. Cancer Sci..

[150] Xia C., He Z., Cai Y., Liang S. (2020). Vorinostat upregulates MICA via the PI3K/Akt pathway to enhance the ability of natural killer cells kill tumor cells. Eur. J. Pharmacol..

[151] Chen L., Jin T., Zhu K., Piao Y., Quan T., Quan C., Lin Z. (2017). PI3K/mTOR dual inhibitor BEZ235 and histone deacetylase inhibitor trichostatin A synergistically exert anti-tumor activity in breast cancer. Oncotarget.

[152] Rahamani M., Aust M.M., Benson E.C., Wallace L., Friedberg J., Grant S. (2014). PI3K/mTOR inhibition markedly potentiates HDAC inhibitor activity in NHL cells through BIM- and MCL-1-dependent mechanisms in vitro and in vivo. Clin. Cancer Res..

[153] Wang Q., Li N., Wang X., Kim M.M., Evers B.M. (2002). Augmentation of sodium butyrate-induced apoptosis by phosphatidylinositol 3-kinase inhibition in the KM20 human colon cancer cell line. Clin. Cancer Res..

[154] Denlinger C.E., Rundall B.K., Jones D.R. (2005). Inhibition of phosphatidylinositol 3-kinase/Akt and histone deacetylase activity induces apoptosis in non-small lung cancer in vitro and in vivo. J. Thorac. Cardiovasc. Surg..

[155] Piao J., Chen L., Quan T., Li L., Quan C., Piao Y., Jin T., Lin Z. (2016). Superior efficacy of co-treatment with the dual PI3K/mTOR inhibitor BEZ235 and histone deacetylase inhibitor Trichostatin A against NSCLC. Oncotarget.

[156] Blumenschein G.R., Kies M.S., Papadimitrakopoulou V.A., Lu C., Kumar A.J., Ricker J.L., Chiao J.H., Chen C., Frankel S.R. (2008). Phase II trial of the histone deacetylase inhibitor Vorinostat (Zolinza, suberoylanilide hydroxamic acid, SAHA) in patients with recurrent and/or metastatic head and neck cancer. Investig. New Drugs.

[157] Erlich R.B., Kherrouche Z., Rickwood D., Endo-Munoz L., Cameron S., Dahler A., Hazar-Rethinam M., de Long L.M., Wooley K., Guminski A. (2012). Preclinical evaluation of dual PI3K-mTOR inhibitors and histone deacetylase inhibitors in head and neck squamous cell carcinoma. Br. J. Cancer.

[158] Meng W., Wang B., Mao W., Wang J., Zhao Y., Li Q., Zhang C., Ma J. (2019). Enhanced efficacy of histone deacetylase inhibitor panobinostat combined with dual PI3K/ mTOR inhibitor BEZ235 against glioblastoma. J. Med. Sci..

[159] Pel Y., Liu K.-W., Wang J., Garancher A., Tao R., Esparza L.A., Maier D.L., Udaka Y.T., Murad N., Morrissy S. (2016). HDAC and PI3K antagonists cooperate to inhibit growth of MYC-driven Medulloblastoma. Cancer Cell.

[160] Chou T.C., Talalay P. (1984). Quantitative analysis of dose-effect relationships: The combined effects of multiple drugs or enzyme inhibitors. Adv. Enzym. Regul..

[161] Alu K.M., Wang A.Z., Park S.I. (2020). Pretargeted delivery of PI3K/mTOR small-molecule inhibitor-loaded nanoparticles for treatment of non-Hodgkin’s lymphoma. Sci. Adv..

